# Serum and Amniotic Fluid Metabolic Profile Changes in Response to Gestational Diabetes Mellitus and the Association with Maternal–Fetal Outcomes

**DOI:** 10.3390/nu13103644

**Published:** 2021-10-18

**Authors:** Yalin Zhou, Runlong Zhao, Ying Lyu, Hanxu Shi, Wanyun Ye, Yuwei Tan, Rui Li, Yajun Xu

**Affiliations:** 1 Department of Nutrition and Food Hygiene, School of Public Health, Peking University, NO.38 Xueyuan Road, Beijing 100083, China; zylyingyang@163.com (Y.Z.); 1510306204@pku.edu.cn (R.Z.); lybjmu@126.com (Y.L.); 903916212@163.com (H.S.); YeWanyun_Vera@bjmu.edu.cn (W.Y.); tanyuwei911@163.com (Y.T.); lr15321657608@163.com (R.L.); 2 PKUHSC-China Feihe Joint Research Institute of Nutrition and Healthy Lifespan Development, NO.38 Xueyuan Road, Beijing 100083, China; 3 Beijing Key Laboratory of Toxicological Research and Risk Assessment for Food Safety, Peking University, NO.38 Xueyuan Road, Beijing 100083, China

**Keywords:** metabolic profiles, gestational diabetes mellitus, untargeted metabolism analysis

## Abstract

This study was designed to identify serum and amniotic fluid (AF) metabolic profile changes in response to gestational diabetes mellitus (GDM) and explore the association with maternal–fetal outcomes. We established the GDM rat models by combining a high-fat diet (HFD) with an injection of low-dose streptozotocin (STZ), detected the fasting plasma glucose (FPG) of pregnant rats in the second and third trimester, and collected AF and fetal rats by cesarean section on gestational day 19 (GD19), as well as measuring the weight and crown–rump length (CRL) of fetal rats. We applied liquid chromatography–tandem mass spectrometry (LC-MS/MS) for the untargeted metabolomics analyses of serum and AF samples and then explored their correlation with maternal–fetal outcomes via the co-occurrence network. The results showed that 91 and 68 metabolites were upregulated and 125 and 78 metabolites were downregulated in serum and AF samples exposed to GDM, respectively. In maternal serum, the obvious alterations emerged in lipids and lipid-like molecules, while there were great changes in carbohydrate and carbohydrate conjugates, followed by amino acids, peptides, and analogs in amniotic fluid. The altered pathways both in serum and AF samples were amino acid, lipid, nucleotide, and vitamin metabolism pathways. In response to GDM, changes in the steroid hormone metabolic pathway occurred in serum, and an altered carbohydrate metabolism pathway was found in AF samples. Among differential metabolites in two kinds of samples, there were 34 common biochemicals shared by serum and AF samples, and a mutual significant association existed. These shared differential metabolites were implicated in several metabolism pathways, including choline, tryptophan, histidine, and nicotinate and nicotinamide metabolism, and among them, N1-methyl-4-pyridone-3-carboxamide, 5’-methylthioadenosine, and kynurenic acid were significantly associated with both maternal FPG and fetal growth. In conclusion, serum and AF metabolic profiles were remarkably altered in response to GDM. N1-Methyl-4-pyridone-3-carboxamide, 5’-methylthioadenosine, and kynurenic acid have the potential to be taken as biomarkers for maternal–fetal health status of GDM. The common and inter-related differential metabolites both in the serum and AF implied the feasibility of predicting fetal health outcomes via detecting the metabolites in maternal serum exposed to GDM.

## 1. Introduction

GDM is defined as the varying degrees of impaired glucose tolerance occurring or first detected during pregnancy and is one of the most common metabolic disorders during pregnancy. It was estimated that 15.8% (20.4 million) of women suffered hyperglycemia during pregnancy and that 83.6% of such cases were due to GDM in 2019 [1]. In China, the prevalence of GDM ranged from 5.3 to 40.2% with an average rate of 14.8% and exhibited an increasing tendency [2,3], greatly threatening mother–child health and placing heavy burdens on the mother–child health care system [4,5]. 

GDM directly affects the intrauterine environment, inducing impaired function of the maternal–fetal unit and disturbed fetal glucose metabolism homeostasis and insulin secretion. In response to the high-glucose intrauterine environment of GDM, the fetus will undergo a series of adaptive alterations such as accelerating the catabolism and utilization of glucose, which promotes its own growth and fat development and results in the high risk of macrosomia and diabetes, obesity, and other chronic metabolic diseases in the adult period [6,7,8].

Pregnant women with GDM typically exhibit increased insulin resistance and reduced insulin secretion, which bring about a succession of abnormal metabolisms that are embodied directly by the aberrant expression of corresponding metabolites. Detecting metabolite profiles of GDM and identifying potential disease-related metabolic pathways will promote the understanding of GDM pathogenesis and ultimately achieve effective prevention and treatment [9]. Additionally, the fetus is directly exposed to AF, which is influenced by GDM [10], so identifying the differential AF metabolites and relevant metabolic pathways involved in the changed intrauterine environment will facilitate the understanding of the mechanism by which GDM has adverse effects on offspring. 

Advances in metabolomics have made it possible to quantify a vast number of metabolites and determine the differential metabolites under the specific disease status. For example, a growing body of evidence supports the great potential of metabolomics in the identification of novel pathways and early biomarkers of insulin resistance and type 2 diabetes [11,12,13]. Similarly, metabolomics has been applied to explore the diagnosis and etiology of GDM. It was reported that women with GDM have remarkably different metabolite expressions in urine and serum or plasm. The levels of metabolites related to amino acid, glycerophospholipid, fatty acid, and steroid hormone metabolism vary between GDM and the healthy condition [14,15,16]. Limited studies have shown that maternal GDM also causes altered metabolic characteristics of AF [10,17]. Since AF can reflect both maternal and fetal health, linking AF metabolic profiles with specific pathological conditions is conducive to discovering biomarkers and better guiding clinical practice. However, there are insufficient studies on metabolite profiles in AF and their association with metabolites in serum from GDM. Therefore, we established GDM animal models, detected the metabolite profiles in serum and AF, and further analyzed the association with maternal–fetal phenotypes in order to identify potential biomarkers indicative of maternal–fetal health outcomes of GDM. 

## 2. Materials and Methods

### 2.1. Establishment of GDM Animal Model

Sprague Dawley (SD) rats were used throughout the study. Female and male rats were obtained from the Department of Laboratory Animal Science of Peking University (Beijing, China, SCXK-2012-0015) and kept in a specific-pathogen-free (SPF) facility with temperature of 22 ± 2 °C, humidity of 50–60%, and 12 h/12 h high/dark cycle. Rats were provided distilled water ad libitum. The experiment had previously been approved by the ethics committee of Peking University (LA2021107).

The combination of HFD with intraperitoneal injection of 30 mg/kg STZ was adopted to establish GDM rat models. Four-week-old female rats were randomly grouped into the control group (CON) and GDM group (GDM) after adaptive feeding for one week. For 8 weeks, the rats in CON were fed with general chow and the rats in GDM were fed with HFD (HFD formula: lard 10%, sucrose 15%, egg yolk powder 15%, casein 5%, cholesterol 1.2%, sodium cholate 0.2%, calcium bicarbonate 0.6%, stone powder 0.4%, rat maintenance feed 52.6%). After that, following fasting for 12 h, the tail vein blood was collected, and the FPG was measured using a rapid blood glucose detector (OneTouch Ultraeasy, LifeScan Inc., Milpitas, CA USA). The female rats with FPG > 6.67 mmol/L were removed due to violating the definition of GDM (FPG levels between 6.67 and 13.89 mmol/L (120–200 mg/dL) were deemed light or mild diabetes [18]). The eligible female rats were mated with 13-week-old healthy male rats overnight at 1:1. The next morning, vaginal plugs and vaginal smear were both used to judge the successful pregnancy. The presence time of vaginal plugs or sperm was recorded as GD0. On GD4, starting at 6:00 p.m., the pregnant rats fasted for 16 h until the next morning. On GD5, the pregnant rats in GDM were intraperitoneally injected with 30 mg/kg of 1% STZ (STZ dissolved in 0.1 mol/L sodium citrate solution, pH = 4.2), and the rats in CON were intraperitoneally injected with 0.1 mol/L sodium citrate solution. Seventy-two hours after injection, the pregnant rats with FPG > 6.67 mmol/L were considered as the successful GDM models. 

### 2.2. Sample Collection and Preparation

The body weight and food intake of pregnant rats were monitored regularly, and abnormal conditions such as lethargy, irritability, immobility, and vaginal bleeding were closely watched. On GD19, the pregnant rats were anesthetized, and the blood and AF samples were collected. The blood was collected from the orbital venous plexus. The AF was collected from the amniotic membrane with a sterile syringe, concentrated in a sterile centrifuge tube, centrifuged at 1000× *g* for 15 min at 4 °C to remove insoluble impurities, and eventually stored at −80 °C. The placenta and fetal rats were aseptically stripped, frozen with liquid nitrogen, and stored at −80 °C for future use. The number, body weight, CRL, and malformations of fetal rats were recorded. 

Fifty microliters of serum sample was transferred to an EP tube. After the addition of 200 μL extract solution (acetonitrile (CNW Technologies, Duesseldorf, Germany)/methanol (CNW Technologies, Duesseldorf, Germany) = 1:1, containing isotopically labeled internal standard mixture), the samples were vortexed for 30 s, sonicated for 10 min in an ice–water bath, and incubated for 1 h at −40 °C to precipitate proteins. Then the sample was centrifuged at 12,000 rpm for 15 min at 4 °C. The resulting supernatant was transferred to a fresh glass vial for analysis. The quality control (QC) sample was prepared by mixing an equal aliquot of the supernatants from all of the samples. AF samples were prepared separately from serum samples but under similar protocols [19].

### 2.3. LC-MS/MS Analysis

The metabolite profiles of serum and AF samples were detected by using an LC-MS/MS system consisting of an ultra-high-performance liquid chromatography (UHPLC) system (Vanquish, Thermo Fisher Scientific, Waltham, MA, USA) and an ultra-high-performance liquid chromatography (UPLC) BEH Amide column (2.1 mm × 100 mm, 1.7 μm) coupled to a Q Exactive (QE) HFX mass spectrometer (Orbitrap MS, Thermo Fisher Scientific, Waltham, MA, USA). The UHPLC system was used for the separation of biochemicals via the UPLC BEH Amide column. The mobile phase consisted of 25 mmol/L ammonium acetate (Sigma-Aldrich, Darmstadt, Germany) and 25 mmol/Lammonia hydroxide (Fisher Chemical, Waltham, MA, USA) in water (pH = 9.75) (A phase) and acetonitrile (B phase). The autosampler temperature was 4 °C, and the injection volume was 3 μL. The QE HFX mass spectrometer was applied for obtaining MS/MS spectra in information-dependent acquisition (IDA) mode in the control of the acquisition software (Xcalibur, Thermo Fisher Scientific, Waltham, MA, USA). In this mode, the acquisition software continuously evaluates the full-scan MS spectrum. The electrospray ion (ESI) conditions were as follows: sheath gas flow rate of 30 Arb, auxiliary gas flow rate of 25 Arb, capillary temperature of 350 °C, full MS resolution of 60,000, MS/MS resolution of 7500, collision energy of 10/30/60 in negative ion mode (NCE), and spray voltage of 3.6 kV (positive) or −3.2 kV (negative).

### 2.4. Statistical Analysis

The original LC-MS/MS data were converted to the mzXML format using ProteoWizard and processed with an in-house program, which was developed using R and based on XCMS, for peak detection, extraction, alignment, and integration. Subsequently, for further multivariate analysis, the raw data were pretreated. Pretreatment included de-noising based on the relative standard deviation (RSD), filling the missing data via half of the minimum value, and normalization by the internal standard normalization method. The final dataset contained the information of peak number, sample name, and normalized peak area and was imported to SIMCA16.0.2 software package (Sartorius Stedim Data Analytics AB, Umea, Västerbotten, Sweden). Firstly, we performed principal component analysis (PCA), an unsupervised analysis, to reduce the dimension of the data. The 95% confidence interval (95% CI) in the PCA score plot was used as the threshold to identify potential outliers in the dataset. Secondly, in order to visualize group separation and find significantly changed metabolites, we conducted the supervised orthogonal projections to latent structures discriminate analysis (OPLS-DA) and acquired the value of variable importance in projection (VIP). The metabolites with VIP > 1 and *p* < 0.05 (*t*-test) were considered as significantly changed metabolites. A 7-fold cross-validation test was performed to evaluate the goodness of fit of the OPLS-DA model by the values of R^2^Y and Q^2^. R^2^Y indicates how well the variation of a variable is explained, and Q^2^ indicates how well a variable can be predicted. A 200-time permutation test was then conducted to assess the robustness of the model. Furthermore, Pearson correlation analysis was adopted for the association between differential metabolites in serum and AF samples. Finally, commercial databases, including Kyoto Encyclopedia of Genes and Genomes (KEGG) (http://www.genome.jp/kegg/ (accessed on 15 June 2021)) and MetaboAnalyst (http://www.metaboanalyst.ca/ (accessed on 15 June 2021)), were used for pathway enrichment analysis. 

Apart from data related to metabolomics, other experimental data were analyzed by using SPSS 22.0 software (SPSS, Inc., Chicago, IL, USA). The data with normal distribution were represented as mean ± standard deviation (Mean ± SD), and differences between groups were compared with *t*-test. In addition, we adopted one-way repeated measures analysis of variance to analyze the data on body weight and food utilization rate of pregnant rats during gestation. All statistical tests were two-tailed, and the differences reached the statistical significance at *p* < 0.05.

## 3. Results

### 3.1. GDM Animal Models 

After 8 weeks of HFD, the body weight was 278.92 ± 20.65 g and 281.6 ± 20.45 g in CON (*n* = 10) and GDM (*n* = 18), respectively, showing no statistical significance (*p* = 0.758). GDM had a slightly higher level of FPG than CON (5.62 ± 0.58 for GDM vs. 5.31 ± 0.54 for CON, *p* = 0.021). After the injection of STZ, eight pregnant rats successfully developed GDM in the GDM group, and two rats aborted in the CON group. Therefore, eight pregnant rats remained in each of the GDM and CON groups. 

**Figure 1:**
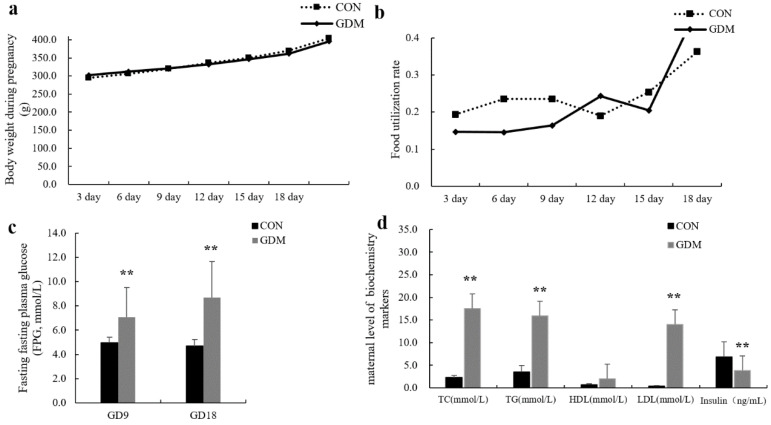
The body weight, food utilization rate, FPG during the gestation, and maternal serum biochemistry markers in CON and GDM. ** *p* < 0.01. (**a**) The line chart of maternal body weight during pregnancy. (**b**) The line chart of maternal food utilization rate during pregnancy. (**c**) The bar chart of fasting plasma glucose in the second and third trimesters. (**d**) The bar chart of maternal level of biochemistry markers.

The body weight of pregnant rats increased with the progression of the pregnancy. Compared with rats in CON, rats with GDM had greater body weight from GD0 to GD9 and a lower weight after GD12 (Figure 1a). The FPG of rats in GDM was significantly higher than that in CON at both GD9 and GD18 (7.06 ± 2.48 mmol/L vs. 4.95 ± 0.47 mmol/L on GD9, *p* < 0.001; 8.68 ± 2.98 mmol/L vs. 4.68 ± 0.54 mmol/L on GD18, *p* < 0.001). The levels of serum total cholesterol (TC), total triglyceride (TG), and low-density lipoprotein (LDL) were significantly greater in GDM, while the insulin concentration of serum was much lower than that in CON (shown in Figure 1a–d), which suggested that the disturbance of glucose and lipid metabolism occurred in rats with GDM.

Table 1 presents the pregnancy outcomes in CON and GDM. In comparison with CON, the GDM group exhibited a greater fetus resorption rate, placental weight, and fetal weight and a smaller number of live fetuses. The greater fetal weight and fewer live fetuses implied the existence of macrosomia and intrauterine growth restriction, respectively. In summary, based on the maternal physical and biochemical indicators and the pregnancy outcomes, the GDM animal models were deemed successful [20].

### 3.2. QC Sample Analysis

QC samples were used to evaluate the stability of the detection system and methods throughout the experiment. All of the correlations of QC samples were close to 1, indicating the acceptable stability and data quality (Figure S1a,b for serum and Figure S2a,b for AF samples). In addition, the isotope was introduced as the internal standard to label the metabolites, and the variations of the response to the internal standard among QS samples were assessed by RSD. RSD ≤15% indicated a stable system and high data quality. For serum samples, RSD was 2.2–2.4% and 4.9–2.8% in positive and negative modes, respectively. For AF samples, RSD was 2.0–2.8% and 3.7–1.1% in positive and negative modes, respectively.

### 3.3. Multivariate Analysis of LC-MS/MS Data

The PCA score plots illustrated an apparent separation of serum and AF samples between GDM and CON, and all of the samples fell within 95% CI with no outliers (Figure S3a,b). The OPLS-DA score plots indicated the significantly different distribution of metabolites in both serum and AF between CON and GDM groups (shown in Figure S4a,b). The OPLS-DA model showed a good degree of explanation of the total variance (R^2^Y = 0.994 for serum samples, R^2^Y = 0.981 for AF samples) and a satisfactory degree of prediction (Q^2^ = 0.837 for serum samples, Q^2^ = 0.927 for AF samples). No overfitting of the models was observed via the permutation test of OPLS-DA (shown in Figure S4c,d).

VIP > 1 and *p* < 0.05 were taken as the cutoff to screen the differentially expressed metabolites. These metabolites were visualized by volcano plots. For serum samples, in the comparison of GDM with CON, the levels of 863 metabolites were reduced, while 971 metabolites were increasingly expressed. For AF samples, comparing GDM with CON, we found that 476 metabolites showed upregulated expression, while the concentrations of 260 metabolites decreased (shown in Figure S4e,f).

### 3.4. Proportion of Identified Metabolites in Serum and Amniotic Fluid

**Figure 2:**
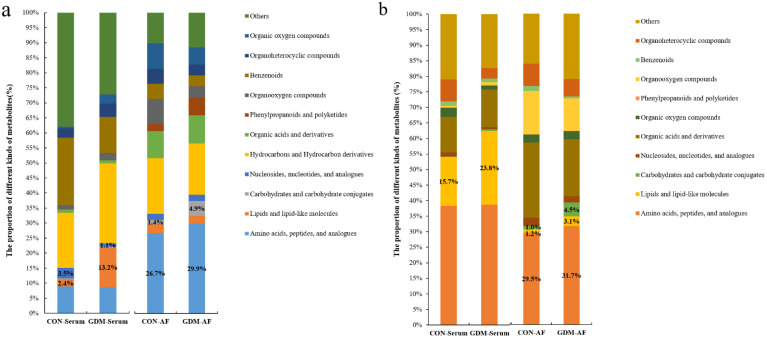
The proportion of different kinds of metabolites detected in serum and AF samples in CON and GDM. (**a**) The proportion of different kinds of metabolites only found in serum or AF samples. (**b**) The proportion of different kinds of common metabolites shared by serum and AF samples. CON-serum: serum samples in CON group; GDM-serum: serum samples in GDM group; CON-AF: AF samples in CON group; GDM-AF: AF samples in GDM group; AF: amniotic fluid.

Based on the accurate mass and MS/MS fragmentation, there were 527 and 595 known metabolites identified in maternal serum and AF samples whether in CON or GDM. A total of 314 metabolites appeared in both serum and AF samples, 281 metabolites existed only in AF, and 213 metabolites were only found in maternal serum. Although the number of metabolites in the GDM and CON groups was the same, there were discrepancies in the composition of metabolites. Among serum-specific metabolites, a remarkably elevated proportion of lipids and lipid-like molecules was observed in GDM (Figure 2a). Among the amniotic fluid-specific metabolites, there were moderately increased proportions of carbohydrates and carbohydrate conjugates, as well as amino acids, peptides, and analogs (Figure 2a). As for the shared metabolites, in maternal serum, moderate alterations appeared in the proportion of lipids and lipid-like molecules, while in AF samples, moderate alterations appeared in the proportion of carbohydrates and carbohydrate conjugates; amino acids, peptides, and analogs; and lipids and lipid-like molecules (Figure 2b).

### 3.5. Identification of Differential Metabolites

All of the biochemicals detected were matched with the online database and were compared between CON and GDM groups. For serum samples, a total of 216 differential metabolites were identified based on the accurate mass and MS/MS fragmentation. In serum exposed to GDM, 91 metabolites were upregulated, while 125 metabolites were downregulated. In AF samples, a total of 146 metabolites were determined: 68 upregulated metabolites and 78 downregulated metabolites. The identification information of these metabolites, including MS/MS score, retention time, the value of *m/z*, superclass in database HMDB, VIP values, and fold change (FC), is detailed in Supplementary Tables S1 and S2 for serum and AF samples, respectively. These biochemicals in serum samples mainly spanned amino acids and derivatives, glycerophospholipids, phosphosphingolipids, steroid hormones, and fatty acids. In AF samples, the differential metabolites mainly covered amino acids and derivatives, glycerophospholipids, phosphosphingolipids, carbohydrates, and carbohydrate conjugates. The differential metabolic profiles are illustrated in the heat map derived from hierarchical clustering analysis (Figure S5a,b for serum samples and AF samples, respectively).

**Figure 3:**
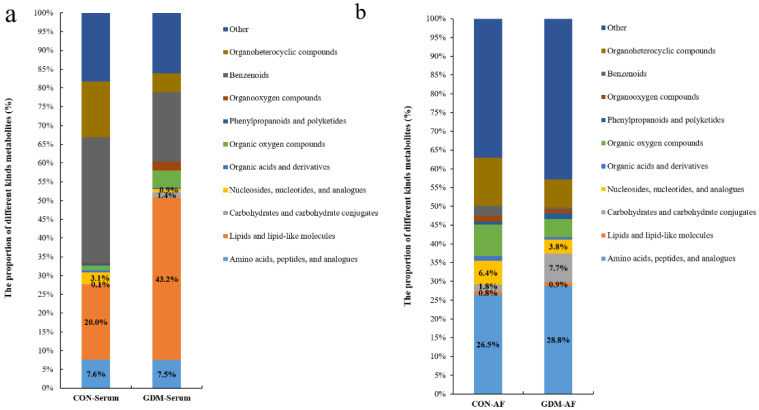
The proportion of differential metabolites in serum and AF samples from CON and GDM. (**a**) The proportion of differential metabolites in serum or AF samples. (**b**) The proportion of differential metabolites in AF samples. CON-serum: serum samples in CON group; GDM-serum: serum samples in GDM group; CON-AF: AF samples in CON group; GDM-AF: AF samples in GDM group; AF: amniotic fluid.

There were disparities in the composition of differential metabolites between CON and GDM groups, as shown in Figure 3a,b. In serum samples, compared with CON, the proportion of lipids and lipid-like molecules in the GDM group significantly increased. However, there was a decreased composition ratio of nucleosides, nucleotides, and analogs. The proportion of metabolites related to amino acids and carbohydrates presented slight alterations (shown in Figure 3a). In AF samples, there was an increased proportion of carbohydrates and carbohydrate conjugates and the moderately elevated composition ratio of amino acids, peptides, and analogs. Conversely, there was a reduced proportion of nucleosides, nucleotides, and analogs. The proportion of lipids and lipid-like molecules almost did not change (shown in Figure 3b).

### 3.6. Altered Metabolic Pathways Induced by Differential Metabolites

**Figure 4:**
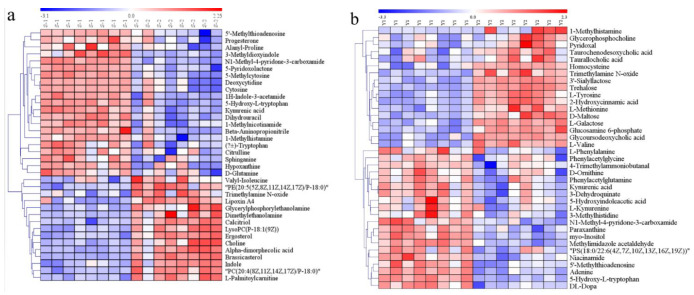
Heat map of differential metabolites analyzed by the hierarchical clustering analysis for serum and AF samples from CON and GDM groups. The horizontal axis represents samples and the vertical axis represents metabolites. Red and blue represent increased and reduced concentrations of metabolites in the GDM group compared with CON. (**a**) The heat map of 36 differential metabolites analyzed by the hierarchical clustering analysis in serum samples. (**b**) The heat map of 37 differential metabolites analyzed by the hierarchical clustering analysis in AF samples. S1: serum samples in CON; S2: serum samples in GDM; Y1: AF samples in CON; Y2: AF samples in GDM.

We matched the differential metabolites with authorized databases such as KEGG and PubChem and found 36 and 37 biochemicals that were involved in varied metabolic pathways in serum and AF samples, respectively. The altered pathways both in serum and AF samples included amino acid metabolism, lipid metabolism, nucleotide metabolism, and vitamin metabolism. The altered pathways related to carbohydrate metabolism were only found in AF samples. These differential metabolites and the corresponding altered pathways are summarized in Table 2 and Table 3 for serum and AF samples, respectively. The expression profiles of differential metabolites implicated in some metabolic pathways are visualized by the heat map of the hierarchical clustering analysis. (Figure 4a,b). 

### 3.7. Differential Metabolites and Their Maternal–Fetal Repercussions

**Figure 5:**
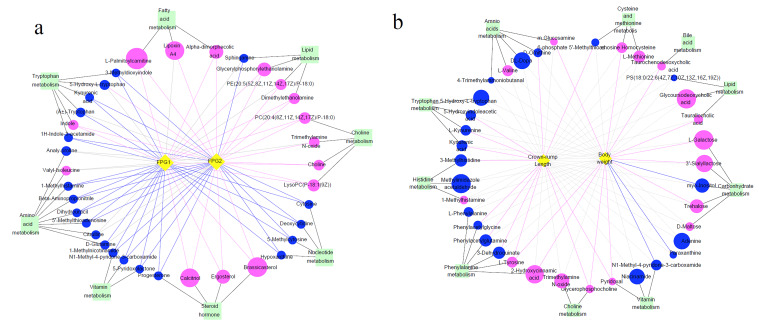
The co-occurrence network of differential metabolites and their maternal–fetal repercussions. The circles: differential metabolites; the diamonds: maternal FPG or fetal body weight or crown–rump length; the squares: the metabolism pathways that metabolites participated in. The blue circles represent the downregulated metabolites compared to CON, and the red circles represent upregulated metabolites. The size of circles means the VIP. Blue lines represent significantly negative associations between metabolites and maternal FPG or fetal body weight or crown–rump length, while the red lines represent significantly positive associations. Grey dotted lines represent no significant association. (**a**) The association of metabolites with maternal FPG during the second and third trimesters. (**b**) The association of metabolites with fetal body weight and crown–rump length. FPG1: fasting plasma glucose in the second trimester; FPG2: fasting plasma glucose in the third trimester.

We further explored the correlation between maternal–fetal phenotypes and distinct metabolites which were engaged in biological pathways. In serum samples, almost all of all metabolites were significantly correlated with maternal FPG in the second and third trimesters. On the whole, FPG was negatively associated with metabolites related to amino acid, nucleotide, and vitamin metabolism pathways, except indole and 5-hydroxy-L-tryptophan, and positively correlated with concentrations of metabolites involved in pathways related to fatty acid, glycerophospholipid, and choline metabolism, as well as steroid metabolism pathways (shown in Figure 5a). As for AF samples, we observed that fetal body weight was positively correlated with the levels of glucosamine 6-phosphate, homocysteine, taurochenodesoxycholic acid, taurallocholic acid, 3’-sialyllactose, L-galactose, trehalose, pyridoxal, glycerophosphocholine, 2-hydroxycinnamic acid, and L-tyrosine, while there was a negative association of fetus body weight with myo-inositol, paraxanthine, and N1-methyl-4-pyridone-3-carboxamide. The metabolites linked to fetal CRL covered pathways related to tryptophan metabolism, phenylalanine metabolism, and vitamin B6 metabolism, as well as cysteine and methionine metabolism (shown in Figure 5b).

**Figure 6:**
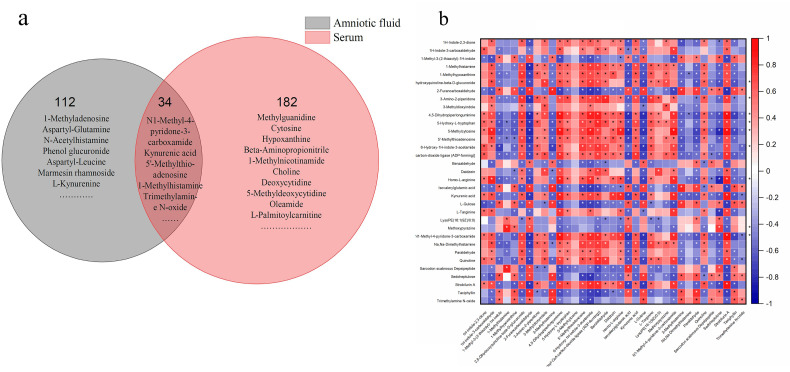
The common differential metabolites in serum and AF samples and their association. (**a**) The Venn diagram of common metabolites in serum and AF samples. (**b**) The correlation heat map of metabolites. The vertical axis: differential metabolites in AF; the horizontal axis: differential metabolites in serum samples. The red represents positive association; the blue represents negative association. The darker the color, the stronger the correlation between the two kinds of samples. The asterisk meant the statistical significance (*p* < 0.05).

**Figure 7:**
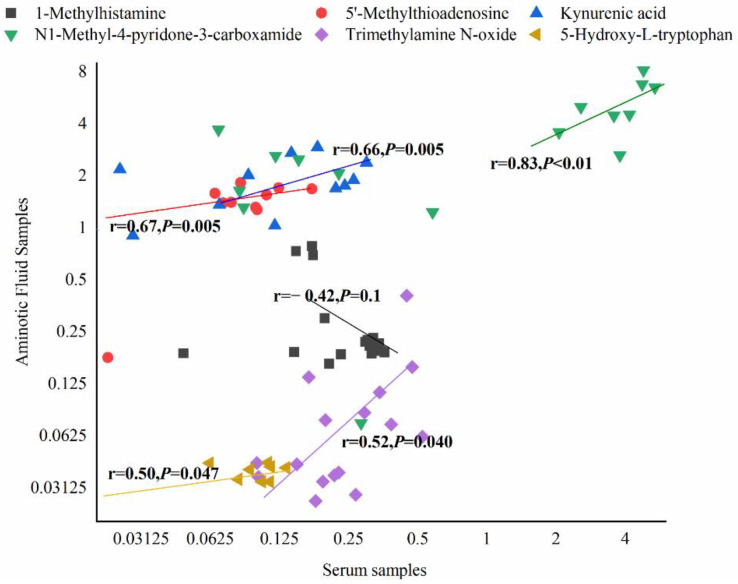
The scatter plots of common metabolites related to maternal or fetal health indicators in serum and AF samples. The horizontal axis: log2 transformation of the concentration of metabolites in serum. The vertical axis: log2 transformation of the concentration of metabolites in AF samples.

Finally, we compared the differential metabolites in serum and AF samples and discovered that they shared 34 common biochemicals (Figure 6a). There was a significant association between these shared metabolites (Figure 6b). Among them, N1-methyl-4-pyridone-3-carboxamide, 1-methylhistamine, trimethylamine N-oxide, 5-hydroxy-tryptophan, kynurenic acid, and 5’-methylthioadenosine participated in some biological metabolism pathways. N1-Methyl-4-pyridone-3-carboxamide was linked with the nicotinate and nicotinamide metabolism, 1-methylhistamine was involved in histidine metabolism, trimethylamine N-oxide was the product of choline metabolism, 5-hydroxy-tryptophan and kynurenic acid participated in tryptophan metabolism, and 5’-methylthioadenosine was implicated in cysteine and methionine metabolism (shown in Table 2 and Table 3). Additionally, 1-methylhistamine was negatively correlated with maternal FPG, while trimethylamine N-oxide and 5-hydroxy-tryptophan had a positive association with maternal FPG in the late trimester. N1-Methyl-4-pyridone-3-carboxamide, 5’-methylthioadenosine, and kynurenic acid were not only negatively linked to maternal FPG, but also presented a positive association with maternal body weight or CRL (shown in Figure 5). Apart from 1-methylhistamine, a significantly positive correlation was found for N1-methyl-4-pyridone-3-carboxamide, trimethylamine N-oxide, 5’-methylthioadenosine, 5-hydroxy-tryptophan, and kynurenic acid in serum and AF samples (Figure 7). 

## 4. Discussion

The serum and amniotic fluid metabolite profiles were remarkably altered in response to GDM. In maternal serum, the obvious alterations emerged in lipids and lipid-like molecules; in amniotic fluid, there were great changes in carbohydrate and carbohydrate conjugates, followed by amino acids, peptides, and analogs. Maternal fasting glucose level was associated with metabolites related to tryptophan, histidine, choline, glycerophospholipid, fatty acid, nucleotide metabolism, and vitamin metabolism pathways. The fetal growth was linked to the metabolites related to tryptophan, histidine, phenylalanine, choline, carbohydrate, and vitamin metabolism in amniotic fluid. There were 34 common metabolites shared by two kinds of samples, and significant correlations existed between them in serum and AF samples. Among these common metabolites, N1-methyl-4-pyridone-3-carboxamide, 5’-methylthioadenosine, and kynurenic acid participated in the regulation of maternal fasting plasma glucose and fetal growth at the same time, underpinning the assumption of predicting maternal–fetal health status via detecting certain metabolites in serum of GDM mothers. It also indicated a parallel mechanism of maternal GDM with abnormal metabolic status of offspring from GDM mothers. 

The common metabolites detected both in maternal serum and amniotic fluid suggested the existence of a selective transport function of the placental barrier and/or some metabolic pathways shared by the maternal and fetal system. However, serum-specific or amniotic fluid-specific metabolites suggested the existence of maternal–fetal metabolic differences since the amniotic fluid is more of a fetal environment. The unique metabolites of amniotic fluid were more abundant right before delivery, indicating that the fetus had become an independent metabolic system. This informed us that the intervention window should be shifted forward in the progeny exposed to adverse environmental factors in early life. 

In the amniotic fluid, the disparities in metabolite composition reflected, to some extent, the changes in placental function caused by GDM. The disturbed intrauterine environment induced by GDM could impair placental development and functions [21,22]. In AF samples exposed to GDM, we found the increased proportions of lipids and lipid-like molecules; carbohydrates and carbohydrate conjugates; and amino acids, peptides, and analogs, and we also observed the elevated level of some metabolites implicated in amino acid, lipid, and carbohydrate metabolic pathways. These findings denoted the increased placental transport of these macronutrients, which might contribute to the larger fetal size and fetal macrosomia.

### 4.1. Differential Metabolites Related to Amino Acid Metabolism in Serum Exposed to GDM

As reported in previous studies, amino acid metabolism has obvious alterations in type 2 diabetes and GDM [14,15]. In parallel, we found in the present research that amino acids and their metabolites changed greatly in serum and amniotic fluid samples of GDM mothers. 

#### 4.1.1. Differential Metabolites Related to Amino Acid Metabolism and Its Relationship with Maternal FPG

Reduced levels of arginine, proline, and tryptophan were found in GDM serum, which was consistent with findings in some existing studies [14,23]. Moreover, in GDM serum samples, we observed a decreased expression of D-glutamine, a precursor of glucose that can effectively stimulate insulin secretion [15]. Its reduction was significantly related to higher maternal plasma glucose in the present study. Conversely, in the Hyperglycemia and Adverse Pregnancy Outcome (HAPO) study, it was found that fasting levels of arginine and proline were positively associated with GDM at 28 weeks’ gestation [24]. In our study, the fasting blood was collected for the metabolome analysis in the third trimester. Variation in gestational weeks might account for the difference. The disparities between our findings and Liu’s hint that the above-mentioned amino acids vary with the progression of pregnancy in GDM. In contrast, there was a significant increase in the serum levels of isoleucine, a branched-chain amino acid (BCAA) that has been widely reported to be associated with increased insulin resistance and the predisposition towards obesity and diabetes [16,25]. In the HAPO study, it was observed that higher levels of BCAAs were not only associated with insulin resistance and GDM but also positively correlated with the later development of a disorder of glucose metabolism [24,26]. Additionally, the elevation of gamma-glutamylisoleucine level suggested the disordered state of the pathway in which glutamyl amino acid is catalyzed to glutathione by gamma-glutamyl transferase. An abnormal glutathione metabolic pathway facilitates hyperglycemia, hyperinsulinemia, insulin resistance, and increased levels of reactive oxygen species and oxidative stress [10]. Consistently, maternal fasting plasma glucose level was negatively linked with some metabolites participating in amino acid metabolism in the present research. 

#### 4.1.2. Changes in Amino Acid Metabolism Profiles and Their Relationship with Fetal Weight and Crown–Rump Length

The altered proportion of metabolites in amniotic fluid could reflect the changes in placental function of transporting materials between maternal–fetal units and fetal metabolic status to a certain extent. In amniotic fluid from GDM, the increased proportion of amino acids and their derivatives indicated the changed amino acid flux through placentas and fetal metabolic processes related to amino acids. 

The levels of phenylalanine and ornithine decreased, while the expression of valine and tyrosine increased. In support of our findings, O’Neill found reduced expression levels of phenylalanine and ornithine in the amniotic fluid of GDM mothers during the second trimester, while the expression levels of leucine and its metabolites increased [10]. As phenylalanines and tyrosine are aromatic amino acids (AAAs), we found that the reduced expression of metabolites related to the phenylalanine metabolism pathway, such as phenylacetylglycine, phenylacetylglutamine, and 3-dehydroquinate, was positively correlated with fetal crown–rump length. Increased levels of L-tyrosine are associated with the future risk of developing type 2 diabetes [27]. In the present research, the level of tyrosine in GDM amniotic fluid increased and was significantly positively correlated with fetal weight. In support of our findings, in the HAPO cohort study, Kadakia et al. found that AAAs (tyrosine) and their metabolic byproducts in cord blood were positively associated with birth weight [28], although we observed no significant association between BCAAs in the amniotic fluid and fetal body weight or crown–rump length. These findings suggested that AAA metabolism might touch upon the pathological process of macrosomia. Valine, a BCAA reported to be correlated with insulin resistance and higher risk of diabetes and obesity, presented an increased expression in our study. It was also reported that the levels of BCAAs and ornithine were increased in cord blood of GDM women [9]. Moreover, the metabolites related to tryptophan, histidine, cysteine, and methionine metabolism played a certain role in the regulation of fetal body weight and CRL, which was also observed in Kadakia’s study [28]. These findings suggest that the disturbed amino acid metabolism might contribute to abnormal outcomes of offspring from mothers with GDM.

### 4.2. Lipid Metabolism

We found remarkable alterations in lipid-related metabolism in GDM serum. Several studies have shown that long-chain fatty acid (LCFA) levels increase in diabetes [12]. Although we observed no LCFAs among differential metabolites, the level of LCFA degradation products was elevated in GDM serum. Fatty acid β-oxidation is one of the critical processes of fatty acid degradation for energy supply. The oxidative products in these pathways aggravate oxidative stress, resulting in impaired pancreas function and insulin secretion [14]. In serum from GDM, we found an increased level of L-palmitoylcarnitine, which was in line with Zhao’s report that the levels of long-chain acylcarnitines were greater in GDM women in both the first and second trimesters [14]. L-Palmitoylcarnitine is an intermediate product of fatty acid oxidation, and its accumulation indicates the emergency of fatty acid peroxidation, which adversely affects insulin secretion [9]. Accordingly, a significantly positive correlation between L-palmitoylcarnitine and maternal FPG was found. Our result was supported by Liu’s findings that the level of long-chain acylcarnitines at 28 weeks’ gestation was positively linked to GDM [26]. In addition, the concentration of lysophosphatidylcholines (LPCs), including LysoPC(18:0), LysoPC(20:0/0:0), and LysoPC(P-18:1(9Z)), was increased and positively associated with maternal FPG. 

As for amniotic fluid samples, unlike the previous study, we found no significantly altered fatty acids or their metabolites [10]. The discrepancy might be explained by the variations in species and the period of sample collection. However, other distinct lipid biochemicals emerged, such as metabolites related to bile acid metabolism. Taurochenodesoxycholic acid is a primary bile acid formed in the liver by conjugation of chenodeoxycholate with taurine, usually in the form of sodium salt [29]. Both glycoudodesoxycholic acid and taurallocholic acid are secondary bile acids and are produced by the colonic microbiota. All of them positively influenced fetal body weight in our study. Bile acids are physiological detergents that promote the excretion, absorption, and transport of fats and sterols in the intestine and liver [30]. Therefore, it has been documented that bile acid exerts crucial impacts on energy metabolism and the incidence of obesity [31]. The elevation in these products related to bile acids indicated accelerated fat degradation, which might adapt to the fat accumulation due to fetal exposure to the high-glucose intrauterine environment. Moreover, the gut-flora-derived secondary bile acids were upregulated, which informed us of the altered microbial composition in the fetus. These findings could provide a better understanding of the role played by bile acid metabolism in the occurrence of macrosomia.

### 4.3. Carbohydrate Metabolism

An elevated carbohydrate proportion with a higher level of hexoses in amniotic fluid might contribute to the increased risk of fetal hyperglycemia. There were elevated concentrations of D-maltose, trehalose, 3′-sialyllactose, and galactose, and 3′-sialyllactose (3′SL) and galactose showed positive correlations with fetal body weight. Galactose is the constituent of lactose in human milk and also one of the core structures of human milk oligosaccharides (HMOs), which are increasingly attracting researchers’ attention because of their critical roles in growth and development and the regulation of the intestinal microbiome [32,33]. Maternal metabolic status has a great impact on the concentration and composition of HMOs. A study reported that a higher level of fasting glucose was observed with a greater serum concentration of 3′-SL in obese women during the first trimester [34]. Additionally, Wise proposed that HMOs came into existence in the amniotic fluid and showed a positive correlation with those in the serum [35]. The increase in 3′-SL in the amniotic fluid exposed to GDM implies a greater level of sialylation, which promotes inflammation and increases susceptibility to type 2 diabetes and cardiovascular diseases [36]. On the other hand, 3′-SL is likely to shape fetal microbiota implantation due to its prebiotic roles and then affect the incidence of long-term diseases [37]. Moreover, galactose is the monomer of HMOs, and its alterations might lead to the disturbance of composition and concentration of some HMOs, which play certain roles in fetal growth and development. To our best knowledge, there are few studies on metabolic profiles of GDM reporting the changes in HMOs.

### 4.4. Common Metabolism in Serum and AF Exposed to GDM

The shared metabolites in serum and amniotic fluid participated in several metabolic pathways, including choline metabolism, tryptophan metabolism, nicotinate and nicotinamide metabolism, histidine metabolism, and methionine metabolism. 

#### 4.4.1. Choline Metabolism

We observed a higher concentration of trimethylamine N-oxide (TMAO) both in serum and amniotic fluid of GDM. TMAO is derived from trimethylamine (TMA), a metabolite of the intestinal microbiota metabolizing choline; TMA is transformed into TMAO under the catalysis of hepatic flavin-containing enzyme monooxygenase 3 (FMO3). It has been documented that the TMAO-related pathway is linked to insulin sensitivity, glucose metabolism, and the high risk of type 2 diabetes [38,39]. The evidence available supported that the reduction in serum TMAO contributed to the improvement of insulin sensitivity among the overweight or obese population [40]. Our findings suggested that the gut-microbe-derived metabolite TMAO could engage in the occurrence of GDM, and the increased TMAO in amniotic fluid might offer an alternative explanation for the susceptibility to metabolic diseases of offspring from GDM mothers. 

#### 4.4.2. Tryptophan Metabolism

Tryptophan is an essential aromatic amino acid and mainly follows two metabolic pathways in host cells, namely the kynurenine and serotonin pathways with the production of kynurenine, kynurenic acid, etc. There is a third metabolic pathway in which gut microbes participate. The gut microbiota directly metabolizes tryptophan into low-molecular-weight materials such as indole and its derivatives, some of which act as aryl hydrocarbon receptor (AhR) ligands [41,42]. We observed that there was a lower level of the tryptophan and its metabolites, included tryptophan, kynurenic acid, 3-methyldioxyindole, and 1H-indole-3-acetamide, in GDM serum. That was in accordance with the previous findings [43,44]. As for amniotic fluid samples from GDM, 5-hydroxyindoleacetic acid, L-kynurenine, and kynurenic acid also presented a reduction compared with CON. However, the increased expression of indole that occurred in serum exposed to GDM implied the non-negligible roles of gut microbiota in the tryptophan metabolism. On the other hand, the reduction in tryptophan in serum induces the insufficient production of AhR derived from gut microbiota decomposing the tryptophan, which brings about a series of metabolic disorders such as insulin resistance and inflammation [41]. 

#### 4.4.3. Histidine Metabolism

1-Methylhistamine is derived from the transformation of histamine catalyzed under the histamine N-methyltransferase. In addition, 1-methylhistamine can be metabolized into methylimidazole acetaldehyde through its interaction with the enzyme amine oxidase [45]. We found that 1-methylhistamine was reduced in the serum of GDM mothers and negatively associated with maternal FPG, which was in accordance with findings in Pan’s study [46]. The decreased level of 1-methylhistamine hinted at an abnormal histidine metabolism and the accumulation of histamine. The former induces the impaired function of scavenging reactive oxygen and nitrogen species [47], and the latter brings about chronic inflammation and disturbances in essential immune response [48], both of which are linked with increased insulin resistance and reduced insulin sensitivity. The elevated concentration of 1-methylhistamine was observed in the amniotic fluid of GDM, while the level of the methylimidazole acetaldehyde, a product of 1-methylhistamine catalyzed by amine oxidase, decreased. The finding implied the occurrence of the inactivated amine oxidase. 1-Methylhistamine is a potentially toxic compound and could inhibit the histaminergic neurons, leading to impaired functions of appetite, wakefulness, emotions, and cognition [49]. In amniotic fluid from GDM, 3-methylhistidine, another metabolite participating in histidine metabolism, was downregulated and positively associated with fetal CRL. The alterations in the corresponding metabolites evidenced the disturbed state of the maternal–fetal histidine metabolism. 

#### 4.4.4. Nicotinate and Nicotinamide Metabolism

Nicotinate and nicotinamide play essential roles in several hundreds of redox reactions in coenzyme form [50]. Additionally, nicotinate, together with trivalent chromium and glutathione, comprises the glucose tolerance factor (GTF) complex, which acts as an insulin cofactor and facilitates the uptake and utilization of glucose. N1-Methyl-4-pyridone-3-carboxamide participates in nicotinate and nicotinamide metabolism. In our study, we observed its reduction both in serum and amniotic fluid samples exposed to GDM, which suggested that the aberrant nicotinate and nicotinamide metabolism occurred in maternal and fetal units. In agreement with our findings, the reduced level of N1-methyl-4-pyridone-3-carboxamide was found in serum from patients with type 2 diabetes [51]. Furthermore, we also observed that maternal FPG and fetal body weight presented an upward trend with the decrease in N1-methyl-4-pyridone-3-carboxamide. There was a significantly positive association between N1-methyl-4-pyridone-3-carboxamide contents in maternal serum and amniotic fluid. 

#### 4.4.5. Methionine Metabolism

5’-Methylthioadenosine (MTA) is involved in two biological pathways, namely the methionine (MET) cycle and spermidine and spermine biosynthesis [29]. MTA is catalyzed into 5-methylthioribose-1-phosphate and adenine by the MTA-phosphorylase, which is a critical reaction in the methionine and purine salvage pathways, respectively. MTA plays a crucial role in regulating gene expression, proliferation, differentiation, and apoptosis. Moreover, MTA shows strong anti-inflammatory effects by inhibiting the circulating proinflammatory cytokines and promoting the synthesis of NO and the production of anti-inflammatory cytokine IL-10 [29,52]. In our study, the downregulation of MTA in maternal serum and amniotic fluid exposed to GDM might mean the abnormality of DNA and protein synthesis and cell energy generation, as well as advanced inflammatory levels. In addition, MTA appeared both in the serum and amniotic fluid and presented significant effects on maternal FPG and fetal growth, which suggested that MTA could be a candidate biomarker for judging maternal–fetal status of GDM. 

These common metabolites existing in serum and amniotic fluid were involved in choline, tryptophan, histidine, nicotinate and nicotinamide, and methionine metabolic pathways, and they had impacts on maternal–fetal health outcomes. Therefore, we proposed the assumption of predicting growth outcomes of fetuses exposed to GDM via testing maternal metabolites, which was supported by the findings reported by Kadakia et al. that the maternal metabolome during pregnancy is associated with fetal growth and adiposity [53]. Moreover, the differentially expressed tryptophan- and choline-related metabolites in serum and amniotic fluid were both closely linked to intestinal microflora, which inspired us to pay attention to the role of intestinal microflora in the pathogenesis of GDM and the increased predisposition to metabolic diseases in offspring of GDM mothers.

## 5. Conclusions

In conclusion, serum and amniotic fluid metabolite profiles were remarkably altered in response to GDM. N1-Methyl-4-pyridone-3-carboxamide, 5’-methylthioadenosine, and kynurenic acid have the potential to be taken as the biomarkers for maternal–fetal health status of GDM. The common and inter-related differential metabolites in the serum and amniotic fluid implied the feasibility of predicting fetal health outcomes via testing the metabolites in maternal serum exposed to GDM.

## Figures and Tables

**Table 1:** The pregnancy outcomes of pregnant rats in CON and GDM groups.

Group	Live Fetus	Resorption Rate	Stillborn Fetus Rate	Plateau Weight (g)	Fetus Weight (g)	CRL (mm)
CON (*n* = 8)	13.7 ± 3.43	1.80% (2/111)	0	0.51 ± 0.07	4.29 ± 0.68	29.01 ± 2.79
GDM (*n* = 8)	10.07 ± 4.58 *	8.49% (9/106) *	1.89% (2/106)	0.72 ± 0.15 **	4.64 ± 0.35 *	29.34 ± 1.48

* *p* <0.05, ** *p* <0.001. CRL: crown–rump length.

**Table 2:** The altered metabolic pathways and the corresponding differential metabolites in serum samples from CON and GDM groups.

Pathway	Metabolites	*p*-Value	FC
Amino Acid Metabolism			
D-Glutamine and D-glutamate metabolism	D-Glutamine	0.010	0.61
Histidine metabolism	1-Methylhistamine	0.001	0.57
β-Alanine metabolism	β-Aminopropionitrile	0.001	0.40
	Dihydrouracil	<0.001	0.27
Cysteine and methionine metabolism	5’-Methylthioadenosine	<0.001	0.20
Arginine and proline metabolism	Citrulline	0.009	0.59
Cyanoamino acid metabolism	β-Aminopropionitrile	0.001	0.40
Isoleucine metabolism	Valyl-isoleucine	0.024	2.13
Proline	Analy-proline	0.0151	0.43
Tryptophan metabolism	(Â±)-Tryptophan	0.0150	0.54
	Kynurenic acid	<0.001	0.12
	3-Methyldioxyindole	0.029	0.02
	1H-Indole−3-acetamide	<0.001	0.22
	Indole	<0.001	1.65
	5-Hydroxy-L-tryptophan	<0.001	0.24
Lipid metabolism			
Glycerophospholipid metabolism	Choline	0.002	2.19
	Dimethylethanolamine	0.016	2.77
	LysoPC(P-18:1(9Z))	0.001	3.76
	PC(20:4(8Z,11Z,14Z,17Z)/P-18:0)	<0.001	3.27
	Glycerylphosphorylethanolamine	0.022	5.60
	PE(20:5(5Z,8Z,11Z,14Z,17Z)/P-18:0)	0.001	2.58
Sphingolipid metabolism	Sphinganine	0.004	0.39
Fatty acid metabolism			
Fatty acid degradation metabolism	L-Palmitoylcarnitine	0.004	13.69
Arachidonic acid metabolism	Lipoxin A4	0.012	10.87
	PC(20:4(8Z,11Z,14Z,17Z)/P-18:0)	<0.001	3.27
Linoleic acid metabolism	α-Dimorphecolic acid	0.001	5.51
	PC(20:4(8Z,11Z,14Z,17Z)/P-18:0)	<0.001	3.27
α-Linolenic acid metabolism	PC(20:4(8Z,11Z,14Z,17Z)/P-18:0)	<0.001	3.27
Steroid hormone			
Steroid biosynthesis	Brassicasterol	<0.001	11.81
	Calcitriol	0.001	10.85
	Ergosterol	<0.001	5.83
Steroid hormone biosynthesis	Progesterone	0.035	0.36
Nucleotide metabolism			
Pyrimidine metabolism	Cytosine	<0.001	0.32
	Deoxycytidine	<0.001	0.30
	5-Methylcytosine	<0.001	0.12
	Dihydrouracil	<0.001	0.27
Purine metabolism	Hypoxanthine	0.007	0.21
Vitamin metabolism			
Nicotinate and nicotinamide metabolism	1-Methylnicotinamide N1-Methyl-4-pyridone-3-carboxamide	<0.001 <0.001	0.28 0.05
Vitamin B6 metabolism	5-Pyridoxolactone	<0.001	0.04
Pantothenate and CoA biosynthesis	Dihydrouracil	<0.001	0.27
Choline metabolism	Choline	0.002	2.19
	Trimethylamine N-oxide	0.004	1.97
	LysoPC(P-18:1(9Z))	0.001	3.76
	PC(20:4(8Z,11Z,14Z,17Z)/P-18:0)	<0.001	3.27

FC: Fold change was derived from normalized means of metabolites in GDM divided by that in CON.

**Table 3:** The altered metabolic pathways and the corresponding differential metabolites in AF samples from CON and GDM groups.

Pathway	Metabolites	*p*-Value	FC
Amino acids metabolism			
Phenylalanine, tyrosine, and tryptophan biosynthesis	L-Phenylalanine	0.047	0.67
	L-Tyrosine	0.002	2.97
	3-Dehydroquinate	0.002	0.45
Phenylalanine metabolism	L-Phenylalanine	0.047	0.67
	L-Tyrosine	0.002	2.97
	Phenylacetylglutamine	0.022	0.53
	Phenylacetylglycine	0.050	0.36
	2-Hydroxycinnamic acid	<0.001	3.07
Cysteine and methionine metabolism	5’-Methylthioadenosine	0.002	0.66
	Homocysteine	0.001	11.46
	L-Methionine	0.008	2.61
Histidine metabolism	1-Methylhistamine	0.031	2.23
	Methylimidazole acetaldehyde	0.000	0.40
	3-Methylhistidine	0.037	0.37
D-Arginine and D-ornithine metabolism	D-Ornithine	0.015	0.67
Valine, leucine, and isoleucine biosynthesis	L-Valine	0.044	1.42
Tryptophan metabolism	5-Hydroxyindoleacetic acid	0.021	0.64
	L-Kynurenine	0.026	0.64
	Kynurenic acid	0.002	0.46
	5-Hydroxy-L-tryptophan	0.001	0.56
Lysine degradation	4-Trimethylammoniobutanal	0.028	0.65
Valine, leucine, and isoleucine degradation	L-Valine	0.044	1.42
Tyrosine metabolism	L-Tyrosine	0.002	2.97
	DL-Dopa		0.06
Carbohydrate metabolism			
Starch and sucrose metabolism	Trehalose	<0.000	8.34
	D-Maltose	0.010	5.40
Amino sugar and nucleotide sugar metabolism	Glucosamine 6-phosphate	0.004	2.00
Galactose metabolism	myo-Inositol	0.000	0.40
	3’-Sialyllactose	0.000	18.10
	L-Galactose		12.95
Nucleotides metabolism			
Aminoacyl-tRNA biosynthesis	L-Phenylalanine	0.047	0.67
	L-Methionine	0.008	2.61
	L-Valine	0.044	1.42
	L-Tyrosine	0.002	2.97
Purine metabolism	Adenine	0.000	0.44
Lipids metabolism			
Glycerophospholipid metabolism	Glycerophosphocholine	0.022	1.77
	PS(18:0/22:6(4Z,7Z,10Z,13Z,16Z,19Z))	0.010	0.53
Vitamin metabolism			
Nicotinate and nicotinamide metabolism	Niacinamide	0.003	0.35
	N1-Methyl-4-pyridone-3-carboxamide	<0.001	0.37
Vitamin B6 metabolism	Pyridoxal	0.042	1.57
Pantothenate and CoA biosynthesis	L-Valine	0.044	1.42
Choline	Trimethylamine N-oxide	0.031	3.64
Bile acid metabolism			
Primary bile acid biosynthesis	Taurochenodesoxycholic acid	0.043	6.67
Others			
Caffeine metabolism	Paraxanthine	<0.001	0.44

FC: Fold change was derived from normalized means of metabolites in GDM divided by that in CON.

## Data Availability

The datasets used during the current study are available from the corresponding author on reasonable request.

## Supplementary Materials

The following are available online at https://www.mdpi.com/article/10.3390/nu13103644/s1, Figure S1a: The correlation between QC samples of serum under the positive mode, Figure S1b: The correlation between QC samples of serum under the negative mode, Figure S2a: The correlation between QC samples of AF under the positive mode, Figure S2b: The correlation between QC samples of AF under the negative mode, Figure S3a: PCA score plots for serum samples from CON and GDM, Figure S3b: PCA score plots for AF samples from CON and GDM, Figure S4a: OPLS-DA score plots of serum samples for CON vs. GDM, Figure S4(b): OPLS-DA score plots of AF samples for CON vs. GDM, Figure S4c: Permutation plot of OPLS-DA model of serum samples, Figure S4d: Permutation plot of OPLS-DA model of AF samples, Figure S4e: Volcano plot of serum samples for CON vs. GDM, Figure S4f: Volcano plot of AF samples for CON vs. GDM, Figure S5a: Heat map of hierarchical clustering analysis for differential metabolites in serum samples from CON and GDM, Figure S5b: Heat map of hierarchical clustering analysis for differential metabolites in AF samples from CON and GDM, Table S1: The identified differential metabolites in serum samples exposed to GDM, Table S2: The identified differential metabolites in AF samples exposed to GDM.
