# Supplementary material for: Serum and Amniotic Fluid Metabolic Profile Changes in Response to Gestational Diabetes Mellitus and the Association with Maternal–Fetal Outcomes
Source: Nutrients. 2021 Oct 18;13(10):3644. doi: 10.3390/nu13103644 (PMC8539410; doi:10.3390/nu13103644)
Supplement: Supplementary file 1 [file nutrients-13-03644-s001.zip › Supplementary files/Table S1.pdf]

Table S1. The identified differential metabolites in serum samples exposed to GDM.

id	MS2 name	MS/MS score	retention time	m/z	SuperClass	VIP	P-value	Fold-change
1	Methylguanidine	0.999995	281.193	74.0717137	Organic nitrogen compounds	1.60	8.93E-05	0.28
2	Cytosine	0.999902615	219.115	112.0506541	Organoheterocyclic compounds	1.37	2.79E-04	0.32
3	L-Gulose	0.999821615	337.396	203.0523076	Organic oxygen compounds	1.64	1.36E-03	20.60
4	Quinoline	0.999567462	45.803	130.0650194	Organoheterocyclic compounds	1.54	4.09E-04	0.29
5	Hypoxanthine	0.999425077	227.2925	137.0458254	Organoheterocyclic compounds	1.26	7.33E-03	0.21
6	Beta-Aminopropionitrile	0.999392154	84.5589	71.06082785	Organic nitrogen compounds	1.53	1.49E-03	0.40
7	1-Methylnicotinamide	0.999108923	326.264	137.0709685	Organoheterocyclic compounds	1.45	4.80E-05	0.28
8	Choline	0.999079	215.628	104.1071366	Organic nitrogen compounds	1.51	1.96E-03	2.19
9	Sarcodon scabrosus Depsipeptide	0.998989385	228.4085	486.2817939	Organic acids and derivatives	1.41	4.07E-03	20.98
10	Kynurenic acid	0.998288	195.169	190.0496361	Organoheterocyclic compounds	1.61	2.12E-04	0.12
11	Deoxycytidine	0.998080231	219.176	228.0973662	Nucleosides, nucleotides, and analogues	1.36	2.95E-04	0.30
12	5-Methyldeoxycytidine	0.996734923	207.86	242.1131164	Nucleosides, nucleotides, and analogues	1.65	1.14E-05	0.12
13	Oleamide	0.995809308	221.999	282.2786613	Lipids and lipid-like molecules	1.58	1.17E-02	33.05
14	L-Palmitoylcarnitine	0.995676692	196.315	400.341031	Lipids and lipid-like molecules	1.59	4.00E-03	13.69
15	Lipoxin A4	0.995199	127.123	375.2133768	Lipids and lipid-like molecules	1.44	1.24E-02	10.87
16	m-Aminobenzoic acid	0.994971846	299.7845	138.0548912	Benzenoids	1.64	3.63E-07	0.48
17	Sedoheptulose	0.994822692	325.134	233.0628876	Organic oxygen compounds	1.56	3.23E-03	3.03
18	N4-Acetylcytidine	0.994268692	169.59	286.102769	Nucleosides, nucleotides, and analogues	1.53	2.61E-05	0.45
19	Daidzein	0.992957846	24.2104	255.0647667	Phenylpropanoids and polyketides	1.67	6.01E-03	0.04
20	Pyrrolidine	0.991739615	297.467	72.08123967	Organoheterocyclic compounds	1.22	6.45E-03	0.48
21	1-Methylhypoxanthine	0.989530692	91.0477	151.0612404	Organoheterocyclic compounds	1.50	2.00E-04	0.29
22	Nervonyl carnitine	0.985965462	168.4455	102.1279383	Organic nitrogen compounds	1.02	3.81E-02	0.80
23	5'-Methylthioadenosine	0.985878462	87.9875	298.0959494	Nucleosides, nucleotides, and analogues	1.19	3.16E-04	0.20
24	3-Methylguanine	0.984247846	195.224	166.072144	Organoheterocyclic compounds	1.28	2.13E-05	0.32
25	Trimethylamine N-oxide	0.983457846	352.44	76.0761842	Organic nitrogen compounds	1.24	3.69E-03	1.97
26	1-Methyl-3-(2-thiazolyl)-1H-indole	0.983014846	243.3095	215.0635517	Organoheterocyclic compounds	1.51	3.36E-03	2.19
27	5-Methylcytosine	0.982753538	207.926	126.0661498	Organoheterocyclic compounds	1.64	7.10E-06	0.12
28	5-Pyridoxolactone	0.979296077	26.5422	166.049755	Organoheterocyclic compounds	1.50	8.00E-05	0.04
29	Androstenol	0.978426462	24.2485	257.2258343	Lipids and lipid-like molecules	1.65	8.49E-04	11.14
30	Sphinganine	0.977049077	58.1705	302.3046362	Organic nitrogen compounds	1.23	1.11E-02	0.39

31	3-Methyldioxyindole	0.976005231	25.3612	164.0704746	Organoheterocyclic compounds	1.50	2.86E-02	0.02
32	Melleolide C	0.975068462	255.034	449.2140129	Lipids and lipid-like molecules	1.49	7.62E-03	13.79
33	Progesterone	0.973820923	135.731	315.2310909	Lipids and lipid-like molecules	1.12	3.52E-02	0.36
34	LysoPE(16:1(9Z)/0:0)	0.970994615	223.609	452.2768847	Lipids and lipid-like molecules	1.43	2.74E-02	12.73
35	(Â±)-Tryptophan	0.969679462	277.717	205.0969888	Organoheterocyclic compounds	1.16	1.51E-02	0.54
36	(+)-2,3-Dihydro-3-methyl-1H-pyrrole	0.967877692	299.689	84.08122004	Organoheterocyclic compounds	1.52	1.02E-04	0.49
37	1-Pyrrolidinecarboxaldehyde	0.967196923	209.959	100.075915	Organoheterocyclic compounds	1.26	1.66E-02	0.30
38	N2,N2-Dimethylguanosine	0.966760923	203.126	312.1294213	Nucleosides, nucleotides, and analogues	1.49	2.38E-03	0.20
39	(2R)-2-Hydroxy-2-methylbutanenitrile	0.964399846	316.982	100.0759524	Organic oxygen compounds	1.46	3.04E-04	0.65
40	Citrulline	0.958464462	404.485	176.102952	Organic acids and derivatives	1.03	9.45E-03	0.59
41	2-Propylpiperidine	0.957739	220.74	128.1432932	Alkaloids and derivatives	1.50	6.87E-05	0.44
42	1H-Pyrrole-2-carboxaldehyde	0.954458462	87.9887	96.04463794	Organic oxygen compounds	1.57	9.08E-06	0.25
43	2-Hydroxypyridine	0.952798385	4.038715	96.0447457	Organoheterocyclic compounds	1.13	2.53E-02	3.28
44	5-Decanoyl-2-nonylpyridine	0.951164231	302.146	360.322955	Organooxygen compounds	1.50	4.76E-03	0.44
45	5-Heneicosyl-1,3-benzenediol	0.949529462	168.446	405.3718027	Benzenoids	1.47	1.23E-05	0.15
46	3,3,5-triiodo-L-thyronine-beta-D-glucuronoside	0.949378154	304.45	132.1019524	Organic acids and derivatives	1.52	6.76E-05	1.97
47	Pyrimidine	0.948702231	49.1987	81.0450915	Organoheterocyclic compounds	1.11	4.41E-04	0.28
48	(E)-Urocanic acid	0.948129	126.134	139.0501374	Organoheterocyclic compounds	1.65	2.41E-04	0.19
49	LysoPE(18:2(9Z,12Z)/0:0)	0.947417077	220.964	478.2917675	Lipids and lipid-like molecules	1.51	2.52E-03	4.84
50	Homo-L-arginine	0.946173154	563.866	189.1345899	Organic acids and derivatives	1.52	3.03E-05	0.27
51	Pentanenitrile	0.940469923	355.736	84.08122552	Organonitrogen compounds	1.26	8.10E-04	0.67
52	Dimethylethanolamine	0.937932077	167.3315	90.09164239	Organonitrogen compounds	1.36	1.57E-02	2.77
53	LysoPE(16:0/0:0)	0.927929615	221.739	454.2920654	Lipids and lipid-like molecules	1.54	1.06E-02	28.67
54	1H-Indole-2,3-dione	0.920970769	46.8531	148.0390715	Organoheterocyclic compounds	1.27	4.26E-03	0.46
55	N-Hexadecanoylpyrrolidine	0.919624692	219.049	310.3096312	Organoheterocyclic compounds	1.66	6.07E-03	33.27
56	Indoxyl sulfate	0.915246769	26.5071	214.0165296	Organic acids and derivatives	1.46	1.47E-03	0.22
57	Brassicasterol	0.913471308	24.2383	381.3508703	Lipids and lipid-like molecules	1.69	4.22E-04	11.81
58	1-Phenyl-1,3-heneicosanedione	0.913456769	196.264	401.3446508	Organic oxygen compounds	1.60	3.68E-03	14.86
59	2-Furancarboxaldehyde	0.912944846	312.363	97.02872092	Organooxygen compounds	1.43	5.20E-04	33.12
60	4,5-Dihydropiperlonguminine	0.911558692	188.273	276.1588326	Organoheterocyclic compounds	1.68	1.69E-05	0.02
61	PA(20:4(5Z,8Z,11Z,14Z)e/2:0)	0.911417308	227.719	487.2853223	Lipids and lipid-like molecules	1.52	4.94E-03	27.18
62	Phenylalanyl-Tryptophan	0.903825308	189.445	352.1646966	Organic acids and derivatives	1.12	1.62E-02	3.72

63	Calcitriol	0.901582077	32.18345	399.324868	Lipids and lipid-like molecules	1.54	1.08E-03	10.85
64	Benzaldehyde	0.900593231	243.635	107.0493924	Benzenoids	1.28	1.10E-02	1.77
65	N-Cyclopropyl-trans-2-cis-6-nonadienamide	0.899823	45.789	194.1536825	Lipids and lipid-like molecules	1.46	1.59E-04	0.56
66	L-Targinine	0.891654231	527.944	189.1345319	Organic acids and derivatives	1.27	3.23E-03	0.43
67	Octadecanamide	0.888294077	127.2695	284.2942488	Lipids and lipid-like molecules	1.59	1.84E-03	8.55
68	N-(2-Methylpropyl)acetamide	0.888093769	225.434	116.1071084	Organic acids and derivatives	1.60	6.47E-04	0.18
69	4-Hydroxy-1H-indole-3-acetonitrile	0.882735077	107.489	173.0707347	Organoheterocyclic compounds	1.53	1.27E-04	0.28
70	1-(5Z,8Z,11Z,14Z-eicosatetraenoyl)-sn-glycero-3-phosphate	0.880826923	217.555	459.2473878	Lipids and lipid-like molecules	1.36	3.15E-04	0.46
71	LysoPE(0:0/18:3(6Z,9Z,12Z))	0.880186923	222.58	476.2746779	Lipids and lipid-like molecules	1.58	1.53E-03	6.81
72	5,6,7,8-Tetrahydroquinoxaline	0.878503154	230.821	135.0916498	Organoheterocyclic compounds	1.42	1.94E-04	0.64
73	N1-Methyl-4-pyridone-3-carboxamide	0.876346769	79.7064	153.0656412	Organoheterocyclic compounds	1.69	3.14E-05	0.05
74	Alpha-dimorphecolic acid	0.873766462	34.4848	279.2314864	Lipids and lipid-like molecules	1.63	9.62E-04	5.51
75	Valyl-Isoleucine	0.872446462	389.34	231.1699409	Organic acids and derivatives	1.17	2.42E-02	2.13
76	Paraldehyde	0.865008385	57.0061	133.0857716	Organoheterocyclic compounds	1.19	2.65E-03	0.40
77	Demethylated antipyrine	0.863893077	68.85685	175.0862702	Organoheterocyclic compounds	1.58	2.03E-05	0.19
78	1H-Indole-3-carboxaldehyde	0.861455692	277.696	146.0598226	Organoheterocyclic compounds	1.17	2.04E-02	0.49
79	1H-Indole-3-acetamide	0.853196462	99.126	175.086299	Organoheterocyclic compounds	1.54	1.54E-04	0.22
80	2,8-Dihydroxyquinoline-beta-D-glucuronide	0.853048923	327.48	338.0863634	Organic oxygen compounds	1.57	1.03E-03	0.03
81	SM(d18:1/16:0)	0.850465692	185.888	703.5728087	Lipids and lipid-like molecules	1.53	1.49E-03	4.60
82	gamma-Calacorene	0.849631077	31.0455	201.1635063	Lipids and lipid-like molecules	1.31	1.71E-03	2.24
83	gamma-Camphorene	0.841993462	25.4206	273.2571015	Lipids and lipid-like molecules	1.69	9.28E-05	6.59
84	Cholesta-4,6-dien-3-one	0.838790077	31.0763	383.3301639	Lipids and lipid-like molecules	1.66	2.88E-04	12.10
85	LysoPC(P-18:1(9Z))	0.834860538	206.574	506.3595385	Lipids and lipid-like molecules	1.62	5.31E-04	3.76
86	3-Amino-2-piperidone	0.833765846	281.174	115.0867154	Organic acids and derivatives	1.40	4.50E-04	0.68
87	2-acetyl-1-alkyl-sn-glycero-3-phosphocholine	0.831339923	227.313	524.3709914	Lipids and lipid-like molecules	1.63	3.34E-03	7.73
88	Isovalerylglutamic acid	0.831285615	262.3105	232.1176455	Organic acids and derivatives	1.53	1.56E-03	4.37
89	Ergosterol	0.825476692	25.9442	379.3349126	Lipids and lipid-like molecules	1.64	2.70E-04	5.83
90	Dihydrouracil	0.824572385	168.434	115.0502635	Organoheterocyclic compounds	1.52	1.05E-05	0.27
91	Polyoxyethylene (600) monoricinoleate	0.823361615	170.74	341.3042962	Lipids and lipid-like molecules	1.52	3.38E-02	56.25
92	apo-[3-methylcrotonoyl-CoA:carbon-dioxide ligase (ADP-forming)]	0.818812231	328.6625	174.1234825	Organic acids and derivatives	1.51	5.20E-05	0.29
93	7-Methylinosine	0.813356	145.831	283.1030375	Nucleosides, nucleotides, and analogues	1.47	2.69E-04	0.43

94	3,4-Dimethyl-5-pentyl-2-furanheptanoic acid	0.810212	124.026	295.2261677	Lipids and lipid-like molecules	1.13	3.83E-04	0.33
95	1-(beta-D-Ribofuranosyl)-1,4-dihydronicotinamide	0.808622769	166.222	257.1126716	Organic oxygen compounds	1.34	1.12E-03	0.45
96	5-Hydroxy-L-tryptophan	0.807833231	49.1113	221.0916994	Organoheterocyclic compounds	1.55	1.89E-04	0.24
97	PI(18:1(9Z)/18:1(9Z))	0.799139385	211.3335	880.5871985	Lipids and lipid-like molecules	1.30	1.85E-03	0.45
98	6-Chloro-N-(1-methylethyl)-1,3,5-triazine-2,4-diamine	0.794654231	277.717	188.0705327	Organoheterocyclic compounds	1.14	1.88E-02	0.55
99	N6-Acetyl-L-lysine	0.792093308	402.138	189.1232537	Organic acids and derivatives	1.26	1.95E-02	2.08
100	alpha-Tocopherolquinone	0.790224077	168.446	447.382465	Lipids and lipid-like molecules	1.18	6.45E-03	0.56
101	Arecaidine	0.789956692	272.3525	142.0861187	Alkaloids and derivatives	1.43	4.88E-04	0.44
102	LysoPE(15:0/0:0)	0.780889308	226.186	440.276589	Lipids and lipid-like molecules	1.36	4.44E-02	17.47
103	beta-Vatirenene	0.767892	29.9397	203.1790949	Lipids and lipid-like molecules	1.51	3.40E-04	2.98
104	LysoPE(18:1(9Z)/0:0)	0.756564231	219.5555	480.3079114	Lipids and lipid-like molecules	1.58	1.52E-02	17.17
105	Metenamine	0.753368615	324.2895	141.1134235	Organoheterocyclic compounds	1.40	7.49E-04	0.43
106	Brassicanal A	0.747145154	144.453	192.0475476	Organoheterocyclic compounds	1.38	1.36E-03	0.14
107	Elaidic carnitine	0.742956615	193.938	426.3570658	Lipids and lipid-like molecules	1.64	3.37E-03	34.31
108	3-Methylhistamine	0.742869538	251.722	126.1026452	Organic nitrogen compounds	1.22	5.64E-03	1.24
109	PC(20:4(8Z,11Z,14Z,17Z)/P-18:0)	0.738921538	36.7217	794.6029745	Lipids and lipid-like molecules	1.63	2.84E-04	3.27
110	Palmitoylethanolamide	0.732098846	85.5069	300.2889965	Organic acids and derivatives	1.28	4.35E-02	4.06
111	Indole	0.726890538	32.1762	118.0651847	Organoheterocyclic compounds	1.51	5.92E-05	1.65
112	LysoPC(16:1(9Z)/0:0)	0.724801308	217.9045	494.3238703	Lipids and lipid-like molecules	1.36	2.01E-02	3.65
113	LysoPE(0:0/18:0)	0.719326385	218.266	482.3231183	Lipids and lipid-like molecules	1.61	5.60E-03	14.07
114	PC(18:2(9Z,12Z)/P-18:1(11Z))	0.718071846	36.7399	768.5875921	Lipids and lipid-like molecules	1.42	1.48E-03	1.85
115	LysoPC(P-16:0)	0.715635154	207.669	480.3441765	Lipids and lipid-like molecules	1.59	5.39E-04	2.42
116	PC(22:4(7Z,10Z,13Z,16Z)/16:0)	0.714243923	155.867	810.5971052	Lipids and lipid-like molecules	1.66	5.17E-07	1.65
117	D-Glutamine	0.713233846	435.893	147.0764365	Organic acids and derivatives	1.10	9.97E-03	0.61
118	PC(20:2(11Z,14Z)/20:4(5Z,8Z,11Z,14Z))	0.711666	36.7272	834.5968613	Lipids and lipid-like molecules	1.00	2.14E-02	1.37
119	(1S,2S,4R,8S)-p-Menthane-1,2,8,9-tetrol 2-glucoside	0.709329923	65.1054	367.195345	Lipids and lipid-like molecules	1.43	7.13E-03	0.05
120	PC(18:1(11Z)/14:0)	0.709125846	37.85805	732.5516588		1.34	8.35E-04	1.93
121	PC(16:0/P-16:0)	0.708554154	161.595	718.571177	Lipids and lipid-like molecules	1.20	2.57E-02	1.79
122	PC(18:0/15:0)	0.708191308	164.997	748.5831026	Lipids and lipid-like molecules	1.14	2.45E-02	0.45
123	2-O-(5,8,11,14,17-Eicosapentaenoyl)-1-O-hexadecylglycero-3-phosphocholine	0.704340231	152.479	766.5723947	Lipids and lipid-like molecules	1.66	1.41E-04	3.58

124	PC(22:6(4Z,7Z,10Z,13Z,16Z,19Z)/22:6(4Z,7Z,10Z,13Z,16Z,19Z))	0.693827538	148.0185	878.5674091	Lipids and lipid-like molecules	1.41	2.96E-04	2.30
125	PC(22:4(7Z,10Z,13Z,16Z)/15:0)	0.692743308	157.021	796.5820981	Lipids and lipid-like molecules	1.48	5.96E-04	0.40
126	trans-Hexadec-2-enoyl carnitine	0.690292923	197.552	398.3254698	Lipids and lipid-like molecules	1.44	6.64E-03	7.92
127	Arachidyl carnitine	0.686002923	189.451	456.4039635	Lipids and lipid-like molecules	1.63	2.45E-02	219.65
128	PC(18:3(6Z,9Z,12Z)/18:1(11Z))	0.685924692	37.831	782.5669553		1.34	3.93E-04	1.74
129	6-Hydroxy-1H-indole-3-acetamide	0.685238692	46.814	191.0812043	Organoheterocyclic compounds	1.38	2.11E-03	0.52
130	SM(d16:1/24:1(15Z))	0.681709	178.808	785.650702	Organic nitrogen compounds	1.55	5.70E-04	2.74
131	PI(20:2(11Z,14Z)/16:0)	0.680446846	211.443	863.5604471	Lipids and lipid-like molecules	1.10	9.46E-04	0.34
132	Strobilurin A	0.680006231	136.584	259.1323656	Benzenoids	1.58	1.71E-04	0.03
133	PC(18:0/P-16:0)	0.678657923	161.5655	746.6042327	Lipids and lipid-like molecules	1.38	8.09E-04	2.42
134	SM(d18:1/14:0)	0.677164846	203.3005	675.5411706	Lipids and lipid-like molecules	1.53	4.06E-04	2.35
135	Glycerylphosphorylethanolamine	0.674843769	406.7865	216.062859	Organic acids and derivatives	1.46	2.24E-02	5.60
136	PC(18:2(9Z,12Z)/18:0)	0.671486308	160.443	786.5970545	Lipids and lipid-like molecules	1.58	3.73E-05	1.34
137	PC(20:5(5Z,8Z,11Z,14Z,17Z)/P-18:1(11Z))	0.669022077	150.2925	790.5718988	Lipids and lipid-like molecules	1.49	9.01E-04	3.13
138	PC(P-18:1(11Z)/22:6(4Z,7Z,10Z,13Z,16Z,19Z))	0.668571692	149.051	816.5878495	Lipids and lipid-like molecules	1.54	5.10E-04	4.78
139	PC(P-18:1(11Z)/22:5(4Z,7Z,10Z,13Z,16Z))	0.666293077	149.139	818.6035923	Lipids and lipid-like molecules	1.56	3.87E-04	5.16
140	CPA(18:0/0:0)	0.666152846	219.408	421.270269	Lipids and lipid-like molecules	1.67	7.16E-03	46.58
141	PC(22:4(7Z,10Z,13Z,16Z)/P-18:0)	0.663613538	148.001	822.6357501	Lipids and lipid-like molecules	1.41	1.34E-03	5.10
142	gamma-Glutamylleucine	0.658421231	455.4535	261.144089	Organic acids and derivatives	1.46	7.19E-03	3.72
143	PC(16:1(9Z)/16:1(9Z))	0.654124154	60.5434	730.5354405	Lipids and lipid-like molecules	1.36	5.71E-03	0.47
144	PC(22:5(4Z,7Z,10Z,13Z,16Z)/22:6(4Z,7Z,10Z,13Z,16Z,19Z))	0.653788462	147.8935	880.5824213	Lipids and lipid-like molecules	1.18	5.41E-03	2.69
145	23-Acetoxysoladulcidine	0.651724769	189.451	474.3570716	Lipids and lipid-like molecules	1.16	3.51E-02	6.14
146	PC(22:5(7Z,10Z,13Z,16Z,19Z)/20:4(5Z,8Z,11Z,14Z))	0.650944231	150.166	856.581035	Lipids and lipid-like molecules	1.10	6.11E-03	1.62
147	N-acetyltryptophan	0.648136462	204.318	247.107251	Organic acids and derivatives	1.32	2.84E-04	0.18
148	Dioscoretine	0.647123769	197.495	242.1746295	Lipids and lipid-like molecules	1.10	7.38E-05	0.33
149	PC(18:3(6Z,9Z,12Z)/P-18:1(11Z))	0.645744923	36.73095	766.5725099	Lipids and lipid-like molecules	1.49	2.24E-05	3.21
150	PC(24:1(15Z)/18:3(6Z,9Z,12Z))	0.644464077	150.191	866.6593917	Lipids and lipid-like molecules	1.05	3.45E-02	3.22
151	SM(d18:1/18:1(9Z))	0.643458692	198.734	729.5879691	Lipids and lipid-like molecules	1.64	2.98E-04	6.87
152	PC(24:1(15Z)/22:4(7Z,10Z,13Z,16Z))	0.642512154	146.964	920.7070093	Lipids and lipid-like molecules	1.20	3.89E-02	6.80
153	LysoPC(22:0)	0.640671923	206.728	580.4324362	Lipids and lipid-like molecules	1.51	9.79E-04	3.99
154	PC(22:4(7Z,10Z,13Z,16Z)/14:0)	0.638279154	158.182	782.566953		1.20	3.72E-03	1.35

155	PC(24:0/20:5(5Z,8Z,11Z,14Z,17Z))	0.637491615	150.183	892.6753862	Lipids and lipid-like molecules	1.24	1.54E-02	2.85
156	PC(24:1(15Z)/20:5(5Z,8Z,11Z,14Z,17Z))	0.637339692	150.152	890.6600079	Lipids and lipid-like molecules	1.12	2.30E-02	2.56
157	PC(22:6(4Z,7Z,10Z,13Z,16Z,19Z)/15:0)	0.631972846	158.18	792.5504584	Lipids and lipid-like molecules	1.54	6.75E-06	0.36
158	PC(22:4(7Z,10Z,13Z,16Z)/22:5(4Z,7Z,10Z,13Z,16Z))	0.630209769	149.042	884.6130363	Lipids and lipid-like molecules	1.30	3.47E-03	3.76
159	SM(d18:1/20:0)	0.628544231	197.334	759.6348402	Lipids and lipid-like molecules	1.33	1.72E-02	2.98
160	Polyethylene, oxidized	0.628513923	138.793	245.1379252	Lipids and lipid-like molecules	1.04	3.95E-02	0.30
161	PC(22:6(4Z,7Z,10Z,13Z,16Z,19Z)/22:2(13Z,16Z))	0.627940231	149.029	886.6286669	Lipids and lipid-like molecules	1.27	4.31E-03	4.31
162	Methoxypyrazine	0.627622692	373.306	111.0554791	Organoheterocyclic compounds	1.21	1.52E-02	1.51
163	Phenylalanyl-Glycine	0.625940692	277.6765	223.1073118	Organic acids and derivatives	1.06	1.61E-02	1.77
164	PC(22:6(4Z,7Z,10Z,13Z,16Z,19Z)/22:1(13Z))	0.624923923	149.114	888.6435375	Lipids and lipid-like molecules	1.25	2.21E-03	4.26
165	PC(20:4(8Z,11Z,14Z,17Z)/14:0)	0.624237462	160.461	754.5344905		1.04	3.43E-02	0.67
166	PC(20:4(8Z,11Z,14Z,17Z)/15:0)	0.622645462	37.9177	768.5507003	Lipids and lipid-like molecules	1.17	4.40E-03	1.57
167	Ganoderic acid Mj	0.617987692	168.441	545.3830109	Lipids and lipid-like molecules	1.44	7.26E-06	0.12
168	DG(14:0/18:4(6Z,9Z,12Z,15Z)/0:0)	0.615211	31.0633	561.4502323	Lipids and lipid-like molecules	1.54	2.57E-03	11.09
169	Isopropylpyrazine	0.614327154	93.9029	123.0916114	Organoheterocyclic compounds	1.41	1.00E-03	0.61
170	PC(20:3(8Z,11Z,14Z)/15:0)	0.614214	161.574	770.564949	Lipids and lipid-like molecules	1.09	1.31E-02	0.58
171	LysoPC(16:0)	0.611611077	277.763	496.3389362	Lipids and lipid-like molecules	1.32	3.63E-02	6.97
172	Cohibin B	0.608589538	29.9507	577.5182087	Lipids and lipid-like molecules	1.42	1.25E-02	4.04
173	(R)-Dihydromaleimide	0.606035615	143.284	100.0395602	Organoheterocyclic compounds	1.39	2.88E-06	0.28
174	PC(14:0/14:0)	0.604875231	168.443	678.504912	Lipids and lipid-like molecules	1.42	4.46E-03	6.60
175	[12]-Gingerol	0.603554462	198.735	396.3101168	Benzenoids	1.20	1.14E-02	4.06
176	DG(20:5(5Z,8Z,11Z,14Z,17Z)/20:3(5Z,8Z,11Z)/0:0)	0.602053154	29.9242	665.5126685	Lipids and lipid-like molecules	1.34	2.62E-03	2.64
177	PC(24:1(15Z)/22:5(4Z,7Z,10Z,13Z,16Z))	0.597065538	147.884	918.6913197	Lipids and lipid-like molecules	1.00	4.79E-02	3.37
178	Acetaminophen glucuronide	0.593401231	268.377	328.102052	Organic oxygen compounds	1.27	1.53E-04	0.28
179	Taxiphyllin	0.590967	335.711	312.1106988	Organic oxygen compounds	1.31	1.42E-03	4.96
180	Na,Na-Dimethylhistamine	0.576462385	44.67	140.1180606	Organic nitrogen compounds	1.39	4.43E-04	0.61
181	Isoleucyl-Histidine	0.572504769	310.148	269.1602226	Organic acids and derivatives	1.42	5.10E-03	0.21
182	Cohibin A	0.567421615	29.9191	549.4874534	Lipids and lipid-like molecules	1.33	8.13E-03	5.13
183	LysoPC(22:6(4Z,7Z,10Z,13Z,16Z,19Z))	0.562843923	204.294	568.338347	Lipids and lipid-like molecules	1.11	5.79E-03	1.60
184	Threoninyl-Aspartate	0.560447	434.3785	235.092061	Organic acids and derivatives	1.06	2.09E-02	1.11
185	PI(18:1(11Z)/18:3(6Z,9Z,12Z))	0.557623769	209.04	859.5310147	Lipids and lipid-like molecules	1.26	2.03E-03	1.77

186	PC(22:6(4Z,7Z,10Z,13Z,16Z,19Z)/16:1(9Z))	0.538531	157.038	804.5501156	Lipids and lipid-like molecules	1.14	1.08E-02	0.71
187	LysoPC(24:0)	0.537602462	204.238	608.463869	Lipids and lipid-like molecules	1.24	6.16E-03	2.53
188	Persicaxanthin	0.534630692	149.172	385.2728141	Lipids and lipid-like molecules	1.37	6.44E-03	12.24
189	LysoPC(18:1(9Z))	0.526261846	206.813	522.3545736	Lipids and lipid-like molecules	1.34	1.36E-03	2.83
190	LysoPC(24:1(15Z))	0.525929538	205.478	606.4484696	Lipids and lipid-like molecules	1.29	5.37E-03	2.41
191	6,10,14-Trimethyl-5,9,13-pentadecatrien-2-one	0.523689154	32.1762	263.2364256	Lipids and lipid-like molecules	1.56	1.47E-03	3.33
192	2-O-(4,7,10,13,16,19-Docosahexaenoyl)-1-O-hexadecylglycero-3-phosphocholine	0.521834615	150.278	792.587198	Lipids and lipid-like molecules	1.66	1.33E-04	4.66
193	LysoPC(18:0)	0.517723077	313.3985	524.3708768	Lipids and lipid-like molecules	1.66	4.55E-03	66.97
194	Benzoyl glucuronide (Benzoic acid)	0.512721077	321.665	299.0778521	Organic oxygen compounds	1.04	3.59E-02	1.48
195	PC(18:4(6Z,9Z,12Z,15Z)/18:1(11Z))	0.502539923	158.253	780.5511986	Lipids and lipid-like molecules	1.30	3.17E-03	0.56
196	Isoquinoline	0.5	31.05325	130.0650107	Organoheterocyclic compounds	1.26	9.37E-03	0.47
197	cis- and trans-2-Isopropyl-4-methyl-1,3-dioxolane	0.499807692	4.776625	131.106541	Organoheterocyclic compounds	1.00	4.00E-02	45.19
198	1-Methylhistamine	0.487567692	46.81525	126.1025254	Organic nitrogen compounds	1.23	5.09E-04	0.57
199	1,2-Dimethyl-4-(6-methyl-4-heptenyl)-1,3-cyclohexadiene	0.482384385	24.2383	219.2103386	Hydrocarbons	1.61	1.41E-03	7.73
200	1-(Hydroxymethyl)-5,5-dimethyl-2,4-imidazolidinedione	0.476365769	404.485	159.0764142	Organoheterocyclic compounds	1.02	1.55E-02	0.62
201	LysoPC(20:0/0:0)	0.475679615	209.038	552.4016344	Lipids and lipid-like molecules	1.64	6.83E-04	6.17
202	Norambreinolide	0.470682	32.7599	251.2001878	Organoheterocyclic compounds	1.45	6.87E-03	3.41
203	PE(20:5(5Z,8Z,11Z,14Z,17Z)/P-18:0)	0.464526538	150.281	750.5396532	Lipids and lipid-like molecules	1.44	1.10E-03	2.58
204	beta-Cubebene	0.463465462	24.2114	205.1947723	Lipids and lipid-like molecules	1.50	5.77E-04	14.54
205	Genistein	0.441146	322.7795	271.059675	Phenylpropanoids and polyketides	1.28	6.75E-04	0.45
206	Feruloyl-2-hydroxyputrescine	0.439217538	258.6155	281.1490586	Phenylpropanoids and polyketides	1.46	1.02E-02	30.60
207	LysoPE(22:0/0:0)	0.436040846	211.09	538.3860309	Lipids and lipid-like molecules	1.47	6.88E-04	2.25
208	3,5-Dichloro-4-hydroxy-2-methoxy-6-methylbenzoic acid	0.416777385	6.24081	250.9874177	Benzenoids	1.08	3.44E-02	2.03
209	Alanyl-Proline	0.404848615	336.88	187.1075331	Organic acids and derivatives	1.24	1.51E-02	0.43
210	(3beta,5alpha,6beta,9alpha,22E,24R)-23-Methylergosta-7,22-diene-3,5,6,9-tetrol	0.402132154	31.0562	461.3615725	Lipids and lipid-like molecules	1.00	2.81E-02	1.73
211	lysoPC(26:1(5Z))	0.374831308	203.7875	634.478661	Lipids and lipid-like molecules	1.01	1.37E-02	2.56
212	PE(P-18:1(11Z)/22:4(7Z,10Z,13Z,16Z))	0.362773077	150.278	778.5736339	Lipids and lipid-like molecules	1.08	3.14E-02	2.34
213	DG(14:1(9Z)/18:2(9Z,12Z)/0:0)	0.361658385	29.9147	563.4661822	Lipids and lipid-like molecules	1.53	3.10E-03	14.11

214	PE(20:4(8Z,11Z,14Z,17Z)/P-18:0)	0.359638692	151.294	752.5558536	Lipids and lipid-like molecules	1.12	8.58E-03	2.11
215	Camellenodiol	0.313317654	32.1825	443.3510316	Lipids and lipid-like molecules	1.42	2.73E-03	4.09
216	2-Phenylethanol	0.307829038	31.0461	105.0700542	Benzenoids	1.54	6.14E-05	2.02
