# Supplementary material for: Serum and Amniotic Fluid Metabolic Profile Changes in Response to Gestational Diabetes Mellitus and the Association with Maternal–Fetal Outcomes
Source: Nutrients. 2021 Oct 18;13(10):3644. doi: 10.3390/nu13103644 (PMC8539410; doi:10.3390/nu13103644)
Supplement: Supplementary file 1 [file nutrients-13-03644-s001.zip › Supplementary files/Table S2.pdf]

Table S2. The identified differential metabolites in amniotic fluid samples exposed to GDM.

id	MS2 name	MS/MS score	Retention time	mz	SuperClass	VIP	P-value	Fold change
10	Nigakinone	0.99836969	282.161	267.077047	Alkaloids and derivatives	1.63	3.41E-07	0.26
51	Dipropyl sulfide	0.92998315	285.596	119.089533	Organosulfur compounds	1.37	3.96E-02	1.43
80	Tromethamine	0.78232546	308.468	122.08125	Organic compounds	1.41	2.51E-02	3.89
128	(E)-Monocrotophos	0.53054346	110.409	224.066253	Organophosphorus compounds	1.80	2.13E-05	1.59
68	2-Furancarboxaldehyde	0.85712969	311.8865	97.0287721	Organooxygen compounds	2.15	4.18E-05	4730.10
87	Gynocardin	0.75182562	316.385	304.099656	Organooxygen compounds	1.13	1.65E-02	0.74
142	3-Dehydroquinate	0.45050423	195.625	191.053171	Organooxygen compounds	1.44	2.13E-03	0.45
23	Trimethylamine N-oxide	0.97942215	353.4835	76.076198	Organic nitrogen compounds	1.64	3.07E-02	3.64
74	3-Methylhistamine	0.81980977	244.071	126.102649	Organic nitrogen compounds	1.71	2.51E-03	1.99
103	Na,Na-Dimethylhistamine	0.68771638	197.911	140.11827	Organic nitrogen compounds	1.09	2.86E-02	1.31
123	1-Methylhistamine	0.56944085	50.6676	126.102636	Organic nitrogen compounds	1.16	3.07E-02	2.23
133	Labienoxime	0.50804846	669.6805	210.185008	Organic nitrogen compounds	1.17	4.58E-02	1.40
19	5'-Methylthioadenosine	0.98103731	87.3152	298.096462	Nucleosides, nucleotides, and analogues	1.11	1.65E-03	0.66
39	N6-Methyladenosine	0.95432362	302.2005	282.119339	Nucleosides, nucleotides, and analogues	1.18	4.54E-02	1.35
62	1-Methyladenosine	0.88033323	126.527	282.119141	Nucleosides, nucleotides, and analogues	1.62	1.27E-03	0.59
69	5'-Deoxy-5'-(methylsulfinyl)adenosine	0.85678038	235.956	314.091259	Nucleosides, nucleotides, and analogues	1.48	9.77E-04	0.56
72	N6-Carbamoyl-L-threonyladenosine	0.827245	341.9095	413.141239	Nucleosides, nucleotides, and analogues	1.15	1.15E-02	0.64
95	6-Thioxanthine 5'-monophosphate	0.71928962	87.3156	381.026977	Nucleosides, nucleotides, and analogues	1.56	5.77E-08	0.24
13	5-O-Galloylhamamelofuranose	0.99058062	302.789	333.07756	Benzenoids	2.15	3.55E-06	17.10
22	Musanolone E	0.98026277	317.5635	305.083722	Benzenoids	1.50	5.58E-03	4.92
32	3-Methoxyanthranilate	0.96756754	32.27975	168.065475	Benzenoids	1.80	9.31E-04	0.23
34	Benzaldehyde	0.96667177	273.06	107.049352	Benzenoids	1.36	1.65E-02	0.70
76	Acetylpterosin C	0.80230946	62.986	277.143002	Benzenoids	2.04	7.25E-12	0.05
109	Strobilurin A	0.65192323	63.1937	259.132417	Benzenoids	1.86	3.04E-11	0.05
111	4-Acetamido-2-amino-6-nitrotoluene	0.63864885	140.278	210.087016	Benzenoids	1.70	3.87E-02	0.10
112	Acetaminophen	0.63727492	62.0892	152.070372	Benzenoids	1.59	1.39E-03	0.80
118	7-Hydroxymethyl-12-methylbenz[a]anthracene sulfate	0.60468723	297.206	353.083705	Benzenoids	1.81	8.71E-05	0.36
8	Daidzein	0.99896023	24.272	255.064731	Phenylpropanoids and polyketides	1.43	7.34E-03	0.27
44	Kanzonol M	0.946032	50.6679	399.176948	Phenylpropanoids and polyketides	1.09	2.39E-04	0.48

56	(E)-4'-Methylresveratrol 3-glucoside	0.92091762	45.6202	405.155326	Phenylpropanoids and polyketides	1.39	4.53E-05	0.35
61	Biochanin A	0.899335	24.272	285.075365	Phenylpropanoids and polyketides	1.34	2.04E-02	0.30
73	Mollicellin D	0.82634238	52.9251	405.107319	Phenylpropanoids and polyketides	1.63	3.29E-02	0.02
97	Diferuloylputrescine	0.70697138	52.9304	441.201592	Phenylpropanoids and polyketides	1.29	2.77E-02	0.48
127	2-Hydroxycinnamic acid	0.555804	315.283	165.05444	Phenylpropanoids and polyketides	1.89	9.16E-05	3.07
132	1-O-p-Coumaroyl-(b-D-glucose 6-O-sulfate)	0.50877323	311.855	407.066386	Phenylpropanoids and polyketides	1.90	4.47E-07	0.33
141	Neosaxitoxin	0.45703669	321.531	316.136245	Phenylpropanoids and polyketides	1.65	4.78E-04	9.84
144	Marmesin rhamnoside	0.40610731	177.085	393.151007	Phenylpropanoids and polyketides	1.11	1.06E-02	0.64
146	Isoliquiritigenin	0.34049892	313.018	257.080411	Phenylpropanoids and polyketides	2.09	4.32E-10	0.27
4	L-Gulose	0.99975923	302.689	203.052513	Organic oxygen compounds	2.05	9.65E-05	5.44
9	D-Maltose	0.99847869	402.96	365.105151	Organic oxygen compounds	1.17	9.79E-03	5.40
14	Sedoheptulose	0.99056969	300.507	233.062801	Organic oxygen compounds	1.57	2.93E-04	1.64
20	1-Kestose	0.98095831	463.669	527.158144	Organic oxygen compounds	1.52	1.51E-03	12.33
21	Trehalose	0.98083177	426.425	365.105104	Organic oxygen compounds	1.55	3.75E-04	8.34
26	Acetone cyanohydrin	0.97662531	97.66775	86.0604875	Organic oxygen compounds	1.84	1.03E-05	0.72
41	L-Kynurenine	0.94836508	278.755	209.091783	Organic oxygen compounds	1.31	2.57E-02	0.64
64	Phenol glucuronide	0.87477938	331.333	293.06293	Organic oxygen compounds	1.36	6.74E-03	0.62
66	6-Hydroxy-5-methoxyindole glucuronide	0.86871131	303.9275	340.102412	Organic oxygen compounds	1.71	1.28E-03	0.06
70	2,8-Dihydroxyquinoline-beta-D-glucuronide	0.85656662	328.968	338.08653	Organic oxygen compounds	1.84	7.42E-04	0.06
71	1-[(5-Amino-5-carboxypentyl)amino]-1-deoxyfructose	0.84499208	521.39	309.165259	Organic oxygen compounds	1.22	1.15E-02	1.65
82	2-Methyl-3-oxopropanoic acid	0.77518223	369.7005	103.050469	Organic oxygen compounds	1.87	1.05E-03	10.33
86	3'-Sialyllactose	0.75860877	436.116	656.200067	Organic oxygen compounds	1.81	3.16E-04	18.10
90	4-Trimethylammoniobutanal	0.73248477	269.621	130.122567	Organic oxygen compounds	1.00	2.84E-02	0.65
91	Glucosamine 6-phosphate	0.73168854	262.612	260.052747	Organic oxygen compounds	1.08	4.12E-03	2.00
100	myo-Inositol	0.70234738	401.846	203.05268	Organic oxygen compounds	1.59	1.72E-06	0.40
107	L-Galactose	0.66039508	308.472	198.097179	Organic oxygen compounds	1.98	1.05E-04	12.95
135	Rhamnose	0.50406362	294.764	187.057409	Organic oxygen compounds	1.29	2.23E-02	3.62
138	Taxiphyllin	0.49620023	334.867	312.110712	Organic oxygen compounds	1.94	3.09E-04	11.30
1	Foeniculoside VII	1	69.0419	349.182608	Lipids and lipid-like molecules	1.06	3.89E-03	3.24
30	LysoPE(16:1(9Z)/0:0)	0.97184254	223.4705	452.276674	Lipids and lipid-like molecules	1.27	2.95E-02	0.69
33	Withaperuvin H	0.96708962	94.2121	579.25952	Lipids and lipid-like molecules	1.07	6.42E-03	0.33
42	17alpha,21-Dihydroxypregnenolone	0.94799738	57.5266	349.236569	Lipids and lipid-like molecules	1.61	3.90E-04	0.18

75	Glycerophosphocholine	0.81640315	400.75	258.109908	Lipids and lipid-like molecules	1.14	2.17E-02	1.77
84	Glycoursodeoxycholic acid	0.769388	226.8	450.321004	Lipids and lipid-like molecules	1.96	2.54E-05	18.54
88	Tripoxyrollin	0.739552	29.4482	589.481851	Lipids and lipid-like molecules	1.47	2.76E-02	3.35
92	Cohibin C	0.72714162	29.9537	577.518796	Lipids and lipid-like molecules	1.76	2.84E-02	4.47
94	8,12-Epoxy-4(15),7,11-eudesmatrien-1-one	0.72170138	63.1605	231.137674	Lipids and lipid-like molecules	1.89	7.33E-06	0.05
98	Taurallocholic acid	0.70518338	197.899	516.298157	Lipids and lipid-like molecules	1.19	4.46E-02	1.91
99	DG(14:0/20:3(5Z,8Z,11Z)/0:0)	0.70272346	28.90795	591.497981	Lipids and lipid-like molecules	1.48	3.20E-02	3.42
113	PC(20:3(8Z,11Z,14Z)/14:0)	0.63456054	36.907	756.551087	Lipids and lipid-like molecules	1.69	3.58E-02	3.04
114	DG(18:4(6Z,9Z,12Z,15Z)/18:1(11Z)/0:0)	0.62471038	30.0014	615.497318	Lipids and lipid-like molecules	1.80	2.61E-02	6.52
115	LysoSM(d18:1)	0.60871092	227.964	465.344853	Lipids and lipid-like molecules	1.60	1.92E-03	1.64
119	LysoPE(22:4(7Z,10Z,13Z,16Z)/0:0)	0.604477	216.321	530.323734	Lipids and lipid-like molecules	1.33	9.92E-03	0.69
120	Glycerol tripropanoate	0.599784	123.12	261.134183	Lipids and lipid-like molecules	1.36	2.23E-02	0.27
131	PC(22:0/P-16:0)	0.51358454	82.7211	802.675132	Lipids and lipid-like molecules	1.15	3.90E-03	0.50
134	Taurochenodesoxycholic acid	0.50578208	160.73	500.303566	Lipids and lipid-like molecules	1.15	4.35E-02	6.67
137	PC(22:5(7Z,10Z,13Z,16Z,19Z)/18:4(6Z,9Z,12Z,15Z))	0.49943923	157.504	828.547279	Lipids and lipid-like molecules	1.58	3.07E-03	1.88
140	PS(18:0/22:6(4Z,7Z,10Z,13Z,16Z,19Z))	0.47225562	209.412	836.541255	Lipids and lipid-like molecules	1.01	9.73E-03	0.53
145	PE(P-18:1(11Z)/22:6(4Z,7Z,10Z,13Z,16Z,19Z))	0.38438085	151.8075	774.540918	Lipids and lipid-like molecules	1.33	2.65E-02	1.75
2	N1-(2-Hydroxyethyl)flurazepam	1	273.0735	333.078772	Organoheterocyclic compounds	1.29	2.07E-03	6.33
5	Quinoline	0.99970408	45.9835	130.065185	Organoheterocyclic compounds	1.67	7.27E-04	0.66
6	Adenine	0.99957838	163.2655	136.061754	Organoheterocyclic compounds	1.76	7.96E-07	0.44
7	Niacinamide	0.99913069	57.427	123.055344	Organoheterocyclic compounds	1.69	2.68E-03	0.35
12	2,5-Dihydro-2,4-dimethyloxazole	0.99416354	52.92665	100.07597	Organoheterocyclic compounds	1.25	3.04E-02	2.83
15	1-Methylhypoxanthine	0.98843669	90.75925	151.061481	Organoheterocyclic compounds	1.54	3.56E-03	1.53
17	Pyridine	0.98745392	57.4835	80.0498684	Organoheterocyclic compounds	1.55	8.86E-03	0.53
18	Kynurenic acid	0.98733615	195.7	190.049758	Organoheterocyclic compounds	1.33	2.01E-03	0.46
24	3-Methyldioxyindole	0.97877092	25.5038	164.070487	Organoheterocyclic compounds	1.92	9.40E-03	0.07
25	5-Methylcytosine	0.97867015	208.234	126.066317	Organoheterocyclic compounds	1.28	4.62E-02	0.74
27	Leukoaminochrome	0.97632392	89.5983	152.06476	Organoheterocyclic compounds	1.23	2.79E-02	1.75
28	1-Methyl-3-(2-thiazolyl)-1H-indole	0.97435692	223.306	215.063605	Organoheterocyclic compounds	2.02	8.90E-03	9.50
31	1H-Indole-3-carboxaldehyde	0.97103877	60.9326	146.059903	Organoheterocyclic compounds	1.73	5.02E-03	0.48
36	Methoxypyrazine	0.96574254	42.61315	111.055474	Organoheterocyclic compounds	1.15	4.29E-02	0.71
38	Nicotinamide N-oxide	0.96128562	154.077	139.050145	Organoheterocyclic compounds	1.93	4.41E-04	0.19

40	Paraldehyde	0.95088346	57.441	133.085819	Organoheterocyclic compounds	1.71	6.52E-04	3.74
46	Pyridoxal	0.941491	315.281	168.065243	Organoheterocyclic compounds	1.08	4.23E-02	1.57
48	Pterolactam	0.93889538	68.88295	116.070721	Organoheterocyclic compounds	1.70	1.23E-02	5.31
53	4,5-Dihydropiperlonguminine	0.92803192	189.879	276.159062	Organoheterocyclic compounds	2.02	1.55E-05	0.06
54	5-Hydroxy-L-tryptophan	0.92636523	39.1577	221.091751	Organoheterocyclic compounds	1.72	8.52E-04	0.56
55	N1-Methyl-4-pyridone-3-carboxamide	0.92313831	79.2828	153.065805	Organoheterocyclic compounds	1.32	3.00E-04	0.37
59	1H-Indole-2,3-dione	0.90698331	47.2157	148.039268	Organoheterocyclic compounds	1.05	1.71E-02	0.73
77	Di-2-furanylmethane	0.79862154	273.061	149.059598	Organoheterocyclic compounds	1.33	2.04E-02	0.69
81	7-Hydroxy-6-methyl-8-ribityl lumazine	0.776949	62.01015	329.109256	Organoheterocyclic compounds	1.45	7.83E-04	0.44
85	Paraxanthine	0.75968054	401.793	219.026428	Organoheterocyclic compounds	1.06	7.71E-05	0.44
89	2-Benzofurancarboxaldehyde	0.73710538	315.286	147.043955	Organoheterocyclic compounds	1.87	2.27E-04	4.36
102	Squamolone	0.69602262	196.922	129.065938	Organoheterocyclic compounds	1.97	1.43E-04	2.04
104	6-Hydroxy-1H-indole-3-acetamide	0.68608108	35.7274	191.081419	Organoheterocyclic compounds	1.88	4.07E-06	0.52
105	Methylimidazole acetaldehyde	0.68575023	58.622	125.07101	Organoheterocyclic compounds	1.95	2.44E-07	0.40
117	Deoxyeritadenine	0.60549562	266.103	238.093099	Organoheterocyclic compounds	1.45	1.95E-03	0.51
125	5-Hydroxyindoleacetic acid	0.56089031	278.751	192.065202	Organoheterocyclic compounds	1.28	2.10E-02	0.64
129	Conhydrinone	0.52010231	224.9445	142.122656	Organoheterocyclic compounds	1.63	1.92E-03	2.31
143	Questiomycin A	0.43279692	303.9335	213.065011	Organoheterocyclic compounds	2.09	4.12E-04	11.30
3	L-Valine	0.99999215	285.5975	118.086329	Organic acids and derivatives	1.34	4.39E-02	1.42
11	L-Phenylalanine	0.99671123	290.194	166.086233	Organic acids and derivatives	1.24	4.65E-02	0.67
16	L-Methionine	0.98760754	308.551	150.058402	Organic acids and derivatives	1.42	7.57E-03	2.61
29	N-Acetylhistamine	0.97202623	146.037	154.097461	Organic acids and derivatives	1.26	1.45E-02	0.60
35	D-Ornithine	0.96607638	525.023	133.097226	Organic acids and derivatives	1.19	1.47E-02	0.67
37	3-Methylhistidine	0.96259369	390.3355	170.092481	Organic acids and derivatives	1.45	3.75E-02	0.37
43	Acrylamide	0.946332	114.9265	72.0448278	Organic acids and derivatives	1.19	2.63E-06	0.34
45	L-Tyrosine	0.94234469	316.253	182.081227	Organic acids and derivatives	1.33	1.61E-03	2.97
47	Phenylacetylglutamine	0.94038569	237.1055	265.118003	Organic acids and derivatives	1.43	2.18E-02	0.53
49	(+)-threo-2-Amino-3,4-dihydroxybutanoic acid	0.93726008	87.3152	136.061833	Organic acids and derivatives	1.07	4.48E-03	0.69
50	Homocysteine	0.93020462	308.472	136.042571	Organic acids and derivatives	1.32	7.44E-04	11.46
52	N-Acryloylglycine	0.92978154	78.10695	130.049831	Organic acids and derivatives	1.43	3.62E-03	9.40
57	Glutamylvaline	0.91863785	464.846	247.128583	Organic acids and derivatives	1.97	1.53E-04	6.91
58	L-Targinine	0.90828408	527.3615	189.134627	Organic acids and derivatives	1.24	1.70E-02	0.65

60	Homo-L-arginine	0.90464754	591.4965	189.13465	Organic acids and derivatives	1.49	9.25E-03	0.44
63	Sarcodon scabrosus Depsipeptide	0.87795708	228.4785	486.282032	Organic acids and derivatives	1.75	6.91E-03	9.70
65	apo-[3-methylcrotonoyl-CoA:carbon-dioxide ligase (AD	0.87213662	327.937	174.123654	Organic acids and derivatives	1.88	1.39E-04	0.41
67	Isovalerylglutamic acid	0.860615	196.922	232.117632	Organic acids and derivatives	2.02	6.32E-05	13.48
78	Saccharopine	0.78770569	341.695	277.132469	Organic acids and derivatives	1.18	7.13E-03	0.57
79	Phenylacetylglycine	0.78703385	195.7855	194.081064	Organic acids and derivatives	1.19	4.99E-02	0.36
83	N-Alpha-acetyllysine	0.76986692	394.319	189.134298	Organic acids and derivatives	1.27	4.20E-02	1.95
93	3-Amino-2-piperidone	0.72656631	541.5605	115.086725	Organic acids and derivatives	1.20	1.88E-02	0.76
96	N2-Fructopyranosylarginine	0.70824308	519	337.171309	Organic acids and derivatives	1.80	1.16E-04	4.17
101	Vinylacetylglycine	0.69972238	434.9325	144.065658	Organic acids and derivatives	1.04	4.11E-02	1.24
106	Hydroxyprolyl-Leucine	0.66829169	315.255	245.149202	Organic acids and derivatives	1.51	2.36E-02	2.02
108	gamma-Glutamylisoleucine	0.653613	450.445	261.144142	Organic acids and derivatives	2.09	4.24E-05	10.66
110	Aspartyl-Leucine	0.64017931	439.022	247.12857	Organic acids and derivatives	1.26	3.92E-02	0.63
116	Epinephrine sulfate	0.60794023	273.079	264.053737	Organic acids and derivatives	1.38	1.42E-02	0.85
121	Glycyl-Valine	0.58829377	406.423	175.107813	Organic acids and derivatives	1.30	1.08E-02	2.18
122	Ethyl carbamate	0.578633	88.46665	90.0553224	Organic acids and derivatives	1.85	2.67E-05	1.75
124	Leucyl-Threonine	0.56567831	510.656	233.149396	Organic acids and derivatives	1.79	1.30E-03	46.74
126	Histidinyl-Leucine	0.56086485	307.326	269.160352	Organic acids and derivatives	1.72	1.13E-03	0.46
130	Aspartyl-Glutamine	0.51844131	436.11	262.103116	Organic acids and derivatives	1.29	3.17E-02	0.59
136	Nopalinic acid	0.50361523	493.823	263.123283	Organic acids and derivatives	1.85	8.22E-05	5.54
139	DL-Dopa	0.49440715	28.799	198.075873	Organic acids and derivatives	1.79	3.78E-03	0.06
